# *Chromera velia*, Endosymbioses and the Rhodoplex Hypothesis—Plastid Evolution in Cryptophytes, Alveolates, Stramenopiles, and Haptophytes (CASH Lineages) 

**DOI:** 10.1093/gbe/evu043

**Published:** 2014-02-25

**Authors:** Jörn Petersen, Ann-Kathrin Ludewig, Victoria Michael, Boyke Bunk, Michael Jarek, Denis Baurain, Henner Brinkmann

**Affiliations:** ^1^Leibniz-Institut DSMZ–Deutsche Sammlung von Mikroorganismen und Zellkulturen GmbH, Braunschweig, Germany; ^2^Helmholtz-Zentrum für Infektionsforschung GmbH, Braunschweig, Germany; ^3^Département des Sciences de la Vie, Université de Liège, Belgium; ^4^PhytoSYSTEMS, Université de Liège, Belgium; ^5^Centre Robert Cedergren, Département de Biochimie, Université de Montréal, Québec, Canada

**Keywords:** next-generation sequencing, eukaryote-to-eukaryote endosymbioses, horizontal and endosymbiotic gene transfer, chromalveolate hypothesis, long-branch attraction artifacts

## Abstract

The discovery of *Chromera velia*, a free-living photosynthetic relative of apicomplexan pathogens, has provided an unexpected opportunity to study the algal ancestry of malaria parasites. In this work, we compared the molecular footprints of a eukaryote-to-eukaryote endosymbiosis in *C. velia* to their equivalents in peridinin-containing dinoflagellates (PCD) to reevaluate recent claims in favor of a common ancestry of their plastids. To this end, we established the draft genome and a set of full-length cDNA sequences from *C. velia* via next-generation sequencing. We documented the presence of a single *coxI* gene in the mitochondrial genome, which thus represents the genetically most reduced aerobic organelle identified so far, but focused our analyses on five “lucky genes” of the Calvin cycle. These were selected because of their known support for a common origin of complex plastids from cryptophytes, alveolates (represented by PCDs), stramenopiles, and haptophytes (CASH) via a single secondary endosymbiosis with a red alga. As expected, our broadly sampled phylogenies of the nuclear-encoded Calvin cycle markers support a rhodophycean origin for the complex plastid of *Chromera*. However, they also suggest an independent origin of apicomplexan and dinophycean (PCD) plastids via two eukaryote-to-eukaryote endosymbioses. Although at odds with the current view of a common photosynthetic ancestry for alveolates, this conclusion is nonetheless in line with the deviant plastome architecture in dinoflagellates and the morphological paradox of four versus three plastid membranes in the respective lineages. Further support for independent endosymbioses is provided by analysis of five additional markers, four of them involved in the plastid protein import machinery. Finally, we introduce the “rhodoplex hypothesis” as a convenient way to designate evolutionary scenarios where CASH plastids are ultimately the product of a single secondary endosymbiosis with a red alga but were subsequently horizontally spread via higher-order eukaryote-to-eukaryote endosymbioses.

## Introduction

Today, it is commonly accepted that photosynthesis in eukaryotes originated in a single primary endosymbiosis with a cyanobacterium and that the direct descendants of this seminal event are the Plantae, that is, green plants, rhodophytes, and glaucophytes (Rodríguez-Ezpeleta et al. 2005; Gould et al. 2008). Their primary plastids are surrounded by two membranes, whereas plastids of all other photosynthetic taxa have three or four membranes, which are indicative of a distinct origin via higher-order endosymbioses (e.g., secondary or tertiary; Delwiche 1999). The latter entail a fundamentally different level of complexity as both partners are eukaryotes (Stoebe and Maier 2002). Prime examples for this process are cryptophytes and chlorarachniophytes, the plastids of which still harbor the so-called nucleomorphs that correspond to the highly reduced eukaryotic nuclei of the, respectively, red and green algal endosymbionts (Douglas et al. 2001; Gilson et al. 2006). With respect to their complex plastids, cryptophytes, haptophytes, stramenopiles, and peridinin-containing dinoflagellates (PCDs) are representatives of the red lineage and share the photosynthetic pigment chlorophyll *c* (Delwiche 1999). Stramenopiles are a eukaryotic superensemble that comprises morphologically diverse protists like, for example, diatoms, kelp, and golden algae as well as aplastidial oomycetes (including the causative agent of the Irish potato blight *Phytophthora infestans*), while dinoflagellates, apicomplexans, and aplastidial ciliates constitute the superensemble alveolates (Adl et al. 2012). Dinoflagellates exhibit various molecular curiosities with giant nuclear genomes of more than 100 Gb (LaJeunesse et al. 2005) and plastid genomes reduced to minicircles (Zhang et al. 1999). Moreover, they show an astonishing symbiotic promiscuity, as documented by kleptoplastidy (Nishitani et al. 2011) and several cases of higher-order endosymbioses where complex algae (i.e., haptophytes, diatoms, and cryptophytes) have been independently reduced to complex plastids (Chesnick et al. 1997; Tengs et al. 2000; Hackett et al. 2003). The identification of nonphotosynthetic plastids in apicomplexan parasites, such as the malaria agent *Plasmodium falciparum*, initiated a paradigm shift in parasitology and offered new weapons for fighting this scourge of humanity (Wilson et al. 1996; Jomaa et al. 1999). Likewise, the discovery of the apicomplexan alga *Chromera velia* astonished the scientific community (Keeling 2008; Moore et al. 2008; Oborník et al. 2009) because of its key position allowing both the investigation of the photosynthetic origin of malaria parasites and the testing of the common photosynthetic ancestry of alveolates. Intriguingly, chlorophyll *c* is absent from *C. velia* and its relative *Vitrella brassicaformis* CCMP3155 (Oborník et al. 2012), but the sequences of their plastomes clearly support an affiliation to the red lineage, in particular stramenopiles (Janouskovec et al. 2010). On the other hand, the apicomplexan algae possess a nuclear-encoded proteobacterial type II RuBisCo that has functionally replaced the typical plastome-encoded cyanobacterial type I enzyme. Because this replacement has only been previously reported for PCDs (Morse et al. 1995), it argues for a common origin of the plastids of these two alveolate groups (Janouskovec et al. 2010). However, in case of a common origin, the different number of four versus three plastid membranes of apicomplexan and dinoflagellate plastids (Graham and Wilcox 2000; Moore et al. 2008) needs to be explained, because membrane loss has never been observed in strictly vertically evolving lineages (Bodył and Moszczyński 2006). Promising insights have been provided by the comparison of the protein import apparatus across all lineages of complex red plastids surrounded by four membranes, including apicomplexan parasites (Bolte et al. 2011), but such a comparison has not been extended to PCDs.

In spite of the exponential growth of the amount of sequence data from plastid-containing eukaryotes, organismal relationships among cryptophytes, alveolates, stramenopiles, and haptophytes (CASH lineages) are still poorly understood. Photoautotrophy is an attractive life style for heterotrophic eukaryotes, but successful endosymbioses are rare events, even in evolutionary time scales. Cavalier-Smith (1999) developed the so-called chromalveolate hypothesis based on the most parsimonious assumption (Occam’s razor) that all contemporary lineages with complex red plastids are vertical descendants of a host cell that experienced a secondary endosymbiosis with a rhodophyte. Though this elegant scenario has initially been supported by five plastid markers of the Calvin cycle (“lucky genes” [Bapteste et al. 2002; Harper and Keeling 2003; Patron et al. 2004; Petersen et al. 2006; Teich et al. 2007]), CASH host-cell phylogenies have always exhibited conflicting paraphyletic relationships (see, e.g., Burki et al. 2008). Pros and cons of the “Chromalveolates” were discussed controversially (Bodył 2005; Sanchez-Puerta and Delwiche 2008; Lane and Archibald 2008, 2009; Bodył et al. 2009), but one main tenet of the chromalveolate hypothesis, that is, the monophyly of the host-cell lineages, has since been falsified by a rigorous phylogenomic approach (Baurain et al. 2010). To reconcile plastid and host-related phylogenies, we and others (Bachvaroff et al. 2005; Bodył 2005; Teich et al. 2007) proposed higher-order eukaryote-to-eukaryote endosymbioses, as illustrated by the metaphor of the Russian Matryoshka dolls (cover image of Petersen et al. 2006). The idea of successive endosymbioses is currently growing in popularity (Burki et al. 2012), and it is a promising starting point to develop a compelling scenario of plastid evolution.

In this study, we established high-quality cDNA data and a complete draft genome sequence from the apicomplexan alga *C. velia* via next-generation sequencing. After the demise of the chromalveolate hypothesis, the current survey was initiated to reevaluate the widely held assumption of a common photosynthetic origin of apicomplexans and PCDs (null hypothesis). Unfortunately, phylogenomics is not applicable to this issue for three reasons. First, the extremely high evolutionary rate of plastid genes in alveolates (Zhang et al. 1999; Janouskovec et al. 2010) and the small subset of minicircle-encoded dinophycean plastid sequences preclude holistic approaches based on the analysis of complete plastomes. Second, due to the horizontal spread of plastids, vertically evolving host-cell markers are inherently uninformative to study the evolution of these organelles. Third, in the course of endosymbiosis, all essential nuclear-encoded plastid markers were independently transferred into the host-cell nucleus. Upon close examination, genuine endosymbiotic gene transfer (EGT) appears to be the exception rather than the rule, and many genes were in fact recruited horizontally (horizontal gene transfer [HGT]). Accordingly, the corresponding single-gene phylogenies are quite complex and the markers are not amenable to concatenation. However, the successful conversion of an alga into a functional plastid is a multifaceted process that results in a mosaic genetic composition in which each gene transfer is a unique diagnostic event of the evolutionary past of the organisms under study. Here, we compared the specific endosymbiosis-related molecular fingerprints of *Chromera* and PCDs in order to test the null hypothesis of a common photosynthetic ancestry. Specifically, we studied the five “lucky” Calvin cycle markers from *C. velia* (i.e., phosphoribulokinase [PRK], sedoheptulose-1,7-bisphosphatase [SBP], fructose-1,6-bisphosphatase [FBP], fructose-1,6-bisphosphate aldolase [FBA], and glyceraldehyde-3-phosphate dehydrogenase [GAPDH]) and our comprehensive phylogenetic analyses provide strong evidence for a common origin of all CASH plastids, most likely in a single secondary endosymbiosis with a red alga. Moreover, we analyzed the 4-hydroxy-3-methylbut-2-enyl disphosphate reductase (HDR), as well as four essential components of the protein import machinery of the plastid (Der1, Cdc48, TIC20, TIC110) previously identified in cryptophytes, stramenopiles, haptophytes, and apicomplexan parasites (van Dooren et al. 2008; Agrawal et al. 2009; Felsner et al. 2011; Stork et al. 2013). Nevertheless, neither the lucky genes nor the HDR or the essential components of the plastid import machinery support the null hypothesis, hence suggesting that apicomplexans and PCDs might actually have recruited their current plastids independently. This apparent conflict can be solved by hypothesizing higher-order eukaryote-to-eukaryote endosymbioses between (and even within) CASH lineages. For practical considerations, we propose a convenient name for this family of phylogenetic scenarios: the “rhodoplex hypothesis.”

## Materials and Methods

### Algal Cultivation and Isolation of Nucleic Acids

The apicomplexan alga *C. velia* (strain CCAP 1602/1) was obtained from the Scottish Culture Collection of Algae and Protozoa (CCAP) and cultivated in 400 ml L1-medium at 22 °C. The respective 1-l Erlenmeyer flask was shaken with 100 rpm in the New Brunswick Scientific Innova 42 incubator shaker under continuous light. Eight hundred and 2,000 mg of algal material was pesteled in liquid nitrogen for DNA and total RNA isolation. One hundred fifty micrograms of genomic DNA was purified with the DNeasy Plant Mini Kit (Qiagen) and 240 µg of total RNA was isolated with the TRIzol reagent (Gibco BRL). The PolyATract mRNA Isolation System III (Promega) was used for the isolation of 300 ng mRNA.

The axenic dinoflagellate *Prorocentrum minimum* (strain CCMP 1329) was obtained from the Provasoli-Guillard National Center for Marine Algae and Microbiota and cultured without shaking in L1-Si medium at 22 °C. 110 nanograms mRNA was isolated from 900 mg of algal material.

### Library Construction and Illumina Sequencing

The Illumina RNA libraries with a size of 300 bp were prepared from mRNA according to the manufacturer’s instructions (*TrueSeq RNA Sample Prep Guide*). The mRNA was directly fragmented using the Covaris S2 system before first-strand cDNA synthesis. The libraries were tagged with specific adapters, quality controlled with the Bioanalyzer/Qubit, and subsequently transferred to the cluster generation platform. The Illumina Cluster Station hybridized the fragments onto the flow cell and amplified them for sequencing on the Genome Analyzer IIx (GA) and HiSeq 2000. Paired-end sequencing of 100–150 bp was performed with the Illumina machine using clustered template DNA and the robust four-color DNA sequencing-by-synthesis technology.

The Illumina DNA library of 450 bp was prepared according to the manufacturer’s instructions (*Preparing Samples for Paired-End-Sequencing*). The DNA was fragmented using the Covaris S2 system. Adapters were ligated to the DNA fragments and the products were purified and size-selected on a gel before the transfer to the cluster generation platform. The Illumina Cluster Station hybridized the fragments onto the flow cell and amplified them for sequencing on the GA. Paired-end sequencing of 110 bp was performed with the GA, and the fluorescent images were processed to sequences using the Illumina GA Pipeline Analysis software 1.8.

DNA and RNA sequence reads were converted to FASTQ format and de novo assembled with VELVET 1.2.07 (Zerbino and Birney 2008). Sequencing data were controlled for general quality features using the fastq-mcf tool of ea-utils (Aronesty 2011). The assembled contigs were scaffolded and extended using paired-read data with SSPACE (Boetzer et al. 2011).

### Phylogenetic Analyses

The alignments generated by ClustalW (Thompson et al. 1997) were manually refined using the ED option of the MUST program package (Philippe 1993). Gblocks was used to eliminate both highly variable and/or ambiguous portions of the alignments (Talavera and Castresana 2007). Maximum likelihood (ML) analyses were performed with RAxML version 7.2.6 (Stamatakis and Alachiotis 2010) under the LG + F + Γ4 model, based on the LG-matrix of amino acid replacements (Le and Gascuel 2008) with empirical amino acid frequencies and four discrete gamma rates. The estimate of the support for internal nodes via bootstrap analyses (100 replicates) was performed with RAxML version 7.2.6 using the same model and the rapid bootstrap option.

## Results

### Establishment of Full-Length cDNA Sequences from *C. velia*

We cultivated *C. velia* CCAP 1602/1, isolated polyA(+) mRNA and constructed a paired-end cDNA library for Illumina sequencing. The raw data of one 150-bp MiSeq, a half 150-bp GA, and a half 101-bp HiSeq lane were used for de novo cDNA assembly. The resulting transcriptome shotgun assemblies (TSAs = cDNA contigs) reached sizes of more than 10 kb. For example, we managed to identify a 13-kb full-length cDNA sequence with 3,619 amino acids (KC899102) that exhibited the highest similarity to a Not1-domain-containing protein from *Cryptosporidium muris* (XP_002140641). In contrast, all homologous Not1 transcripts from apicomplexan parasites, including those of *Plasmodium*, *Toxoplasma*, and *Babesia*, appear to be partial, which indicates that our new *C. velia* data set is of uncommonly high quality.

**Fig. 1.—:**
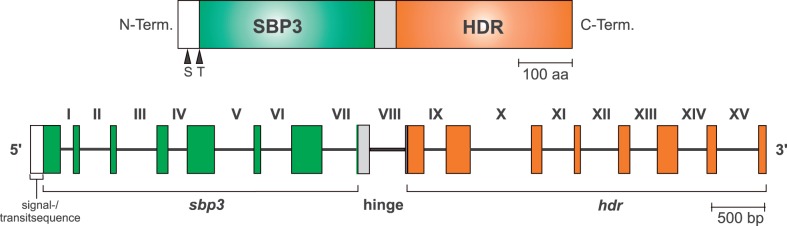
Protein and gene structure of the nuclear-encoded plastid SBP3-HDR fusion protein from *Chromera velia* (KC899090; ARZB00000000). The putative N-terminal cleavage sites of the bipartite signal- and transit peptides are indicated with an “S” and “T,” respectively. A hinge region links the putative Calvin cycle SBP-3 enzyme of *Chromera* (green color) with the HDR enzyme that is essential for the plastid MEP-pathway for isoprenoid biosynthesis (red color). The exon–intron structure of the gene is shown below and introns are indicated with Roman numerals.

The impetus of the present study was to obtain full-length cDNAs for the five lucky genes of the Calvin cycle (PRK, SBP, FBP, FBA, and GAPDH) that are known to support a common origin of complex plastids from the CASH lineages. To this end, we mined our TSAs using sequences from the diatom *Phaeodactylum tricornutum* as queries. Altogether, our TBlastN searches returned 15 cDNAs: a single TSA for each of the Calvin cycle-specific PRK and class II FBA, 2 TSAs for GAPDH, 3 for SBP, 4 for FBP, and 4 for class I FBA (supplementary table S1, Supplementary Material online). The relative expression rate ranged from about 100-fold coverage for the class I aldolase (FBA-Ic; KC899097) to 13,500-fold coverage for the plastid FBP (FBPpla; KC899094). Notably, all but two TSAs comprised the complete protein-encoding region and thus corresponded to full-length cDNAs. The exceptions are two class I aldolases (FBA-Ic [KC899097], FBA-Id [KC899098]), for which the complete cDNAs were assembled from overlapping TSAs. The most conspicuous transcript found in the present study was a cDNA encoding two enzymes of the plastid primary metabolism. The corresponding biprotein consists of an N-terminal SBP and a C-terminal HDR (fig. 1), catalyzing the final step of isoprenoid (isopentenyl pyrophosphate) biosynthesis in the plastid (Grauvogel and Petersen 2007). The two proteins are connected by a hinge region of ∼40 amino acids. We can exclude that the bicistronic *sbp3-hdr* TSA is an assembly artifact, because it perfectly matches the corresponding genomic contigs (KC899090, see later).

### Comparison of *C. velia* cDNAs with Draft Genome Sequences

Genomic DNA from *C. velia* CCAP 1602/1 was also used for Illumina sequencing (GA) and a draft genome assembly was established (BioProject: PRJNA196886). The Whole Genome Shotgun project has been deposited at DDBJ/EMBL/GenBank under the accession ARZB00000000. The version described in this article is the first version, ARZB01000000. The deposited data set has been classified as an algal metagenome because *C. velia* strains are not axenic. However, concomitant bacterial contigs were easy to detect and did not interfere with our analyses. We identified the genuine genomic contigs of all cDNA sequences presented in supplementary table S1, Supplementary Material online. Observed coverage ranged between 16.5 and 20.2 and should be representative for the complete genome of *C. velia*. Moreover, we could identify six overlapping contigs corresponding to the published plastome sequence (nodes 26917, 734, 12638, 190961, 4719, 1132 versus HM222967; Janouskovec et al. 2010) and five contigs corresponding to the complete mitochondrial genome of *C. velia* (KC899110; supplementary fig. S1, Supplementary Material online). The mitochondrial genome has a length of about 2 kb and is circular, as demonstrated by polymerase chain reaction (PCR) and inverse PCR. It contains only a single *coxI* gene encoding the subunit I of the cytochrome c oxidase (EC 1.9.3.1), the last enzyme of the mitochondrial respiratory electron transport chain (complex IV). Sequence polymorphisms in the noncoding region of cloned iPCR amplicons indicate that the mitochondrial genome of *Chromera* is represented by more than one *coxI*-containing minicircle (supplementary fig. S1, Supplementary Material online).

The comparison of cDNA and genomic sequences demonstrated that the 15 novel cDNAs were authentic eukaryotic sequences (KC899087 to KC899101), because it revealed the presence of at least one intron in all but two of the corresponding genes. Up to 32 introns were identified in a single gene (*not*1, supplementary table S1, Supplementary Material online), whereas no intron was found in the plastid GAPDH (*gapC*-I) or in one of the aldolase (*fba*-Id) genes. The first seven introns in the *sbp3-hdr* fusion gene are located within the N-terminal SBP while the last seven introns are part of the C-terminal HDR (fig. 1). The remaining intron (VIII) lies in the connecting hinge region that exhibits no homology to SBP or HDR sequences. Two genomic contigs of our de novo*-*assembled draft genome with lengths of 3 and 8 kb cover the *sbp3-hdr* gene, but a complete assembly of the Illumina sequences was hampered by repetitive sequence motifs in intron XV. Though the size of the latter (∼350 bp) could be estimated by a bridging PCR amplicon (fig. 1), even conventional Sanger sequencing failed due to sequence repeats. As a comparable situation of fragmented gene assemblies was observed for 10 of the 14 intron-containing genes analyzed in the present study, this indicates that repetitive sequence elements are common in the *C. velia* genome.

The availability of the *C. velia* draft genome allowed us to search for potentially nonexpressed genes. Hence, we performed TBlastN searches using diatom plastid class II aldolases as queries (FbaC1: EEC428359, FbaC2: EEC44953; Allen et al. 2012) but could not identify any class II aldolase beyond the aforementioned cytosolic copy (KC899099). Plastid *fba*-II genes are thus missing from the *C. velia* genome. Further searches for all the markers examined in the present study did not reveal additional nonexpressed genes. Therefore, we conclude that our 15 cDNA clones encompass all homologous copies of the five lucky genes encoded in the genome of this apicomplexan alga.

### Phylogenetic Analysis of PRK Sequences

**Fig. 2.—:**
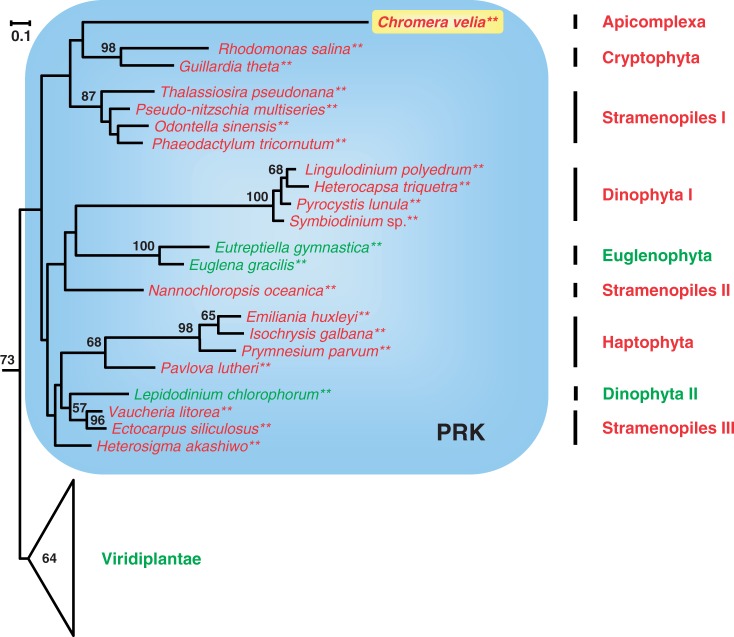
Image detail of a phylogenetic ML analysis of PRK sequences focused on complex algae of the CASH lineages (blue box) and the novel sequence of *C. velia* (highlighted in yellow; KC899087). The complete RAxML analysis was performed with a LG + F + Γ4 model of 65 PRK sequences based on 247 amino acid positions. The tree is rooted on a cyanobacterial outgroup (see supplementary fig. S2*a*, Supplementary Material online). Species names are shown in red and green according to the rhodophycean or chlorophycean origin of their complex plastids.

The PRK is the only Calvin cycle marker of the present study that is encoded by a single gene in the *C. velia* genome and that has no paralogous enzymes involved in glycolysis or gluconeogenesis. We searched for homologous sequences from rhodophytes, glaucophytes, green plants, and all lineages containing complex plastids in the public databases and could identify 11 new PRKs from CASH that had not been analyzed in our previous study (Petersen et al. 2006). The phylogenetic tree is rooted with cyanobacterial sequences, which represent the PRK donor at the origin of the EGT that followed the primary endosymbiosis (supplementary fig. S2*a*, Supplementary Material online; subtrees that have previously provided evidence for a common origin of CASH plastids are highlighted with blue boxes throughout the manuscript). The three eukaryotic lineages with primary plastids, that is, rhodophytes, green plants, and glaucophytes, form distinct subtrees. Because the inclusion of glaucophytes in the analysis does not affect the close affiliation of CASH PRKs to those of green plants (fig. 2, supplementary fig. S2*a*, Supplementary Material online), our former designation “green PRK” is still valid (Petersen et al. 2006). In principle, the currently available diversity of chlorophytes should allow us to pinpoint the putative donor lineage of the green CASH gene, but the CASH subtree emerged basal to all green plants. Suspecting an artifact due to long-branch attraction (LBA; Brinkmann et al. 2005), we reanalyzed our data set after discarding the cyanobacterial outgroup and all complex algae, except the three most slowly evolving stramenopiles (supplementary fig. S2*b*, Supplementary Material online). As expected, the new tree showed stramenopiles deeply nested within Viridiplantae (100% bootstrap proportion [BP]), now emerging as the sister group of Mamiellales (*Ostreococcus, Micromonas*). Statistical support for this position even increased from 73% to 83% after discarding the more distant rhodophyte and glaucophyte outgroups (compare supplementary fig. S2*b* and *c*, Supplementary Material online). The common branch at the base of these stramenopiles is extremely long and characteristic of xenologous genes acquired either by HGT or by EGT subsequent to endosymbiosis. It is generally interpreted as reflecting the period of relaxed selective pressure affecting a xenologous gene between its transfer into the nucleus of the host cell and its recruitment as a functional substitute for the genuine equivalent (either plastid- or nucleomorph-encoded).

Our comprehensive phylogenetic analyses revealed several additional HGTs beyond the green PRK of the CASH lineages. With respect to algal lineages with complex green plastids, euglenophytes and the green dinoflagellate *Lepidodinium chlorophorum* (Matsumoto et al. 2011) obtained their PRKs horizontally from the CASH lineages, whereas chlorarachniophycean PRKs appear to be of red algal origin (supplementary fig. S2*a*, Supplementary Material online). Euglenophytes and chlorarachniophytes do exhibit both long basal branches and a maximal statistical support, which indicates that PRK recruitments occurred early in the history of these lineages. Despite their nonendosymbiotic context, these two HGTs represent unique diagnostic events of each respective secondary endosymbiosis. Similarly, in the current study, we analyzed the phylogenies of the five lucky genes with the aim of finding the same type of unifying support for the null hypothesis of a common origin of the plastid in *Chromera* (representing the apicomplexans) and PCDs. However, with respect to the PRK, only some lineage-specific branches of complex algae with red plastids are highly supported, for example, those of cryptophytes (98% BP) and PCDs (100% BP). Haptophytes are also monophyletic, but the low statistical support (68% BP) reflects the much deeper divergence of their two constitutive lineages Pavlovales and Prymnesiales (including *Emiliania huxleyi*; see also GAPDH below). Stramenopiles are not recovered as a single superensemble, but their monophyly cannot be excluded due to the lack of statistical support observed for deeper phylogenetic relationships. All PRK sequences of alveolates are located in the CASH subtree, but *Chromera* and PCDs do not exhibit the expected sister-group relationship. Interestingly, these two groups do not cluster together, whereas they feature by far the longest branches of this tree (fig. 2, blue box). Thus, even with the helping hand of LBA (Rodriguez-Ezpeleta et al. 2007), the PRK phylogeny does not support the null hypothesis of a common photosynthetic ancestry of apicomplexans and dinoflagellates.

### Phylogenetic Analyses of SBP and FBP Sequences

**Fig. 3.—:**
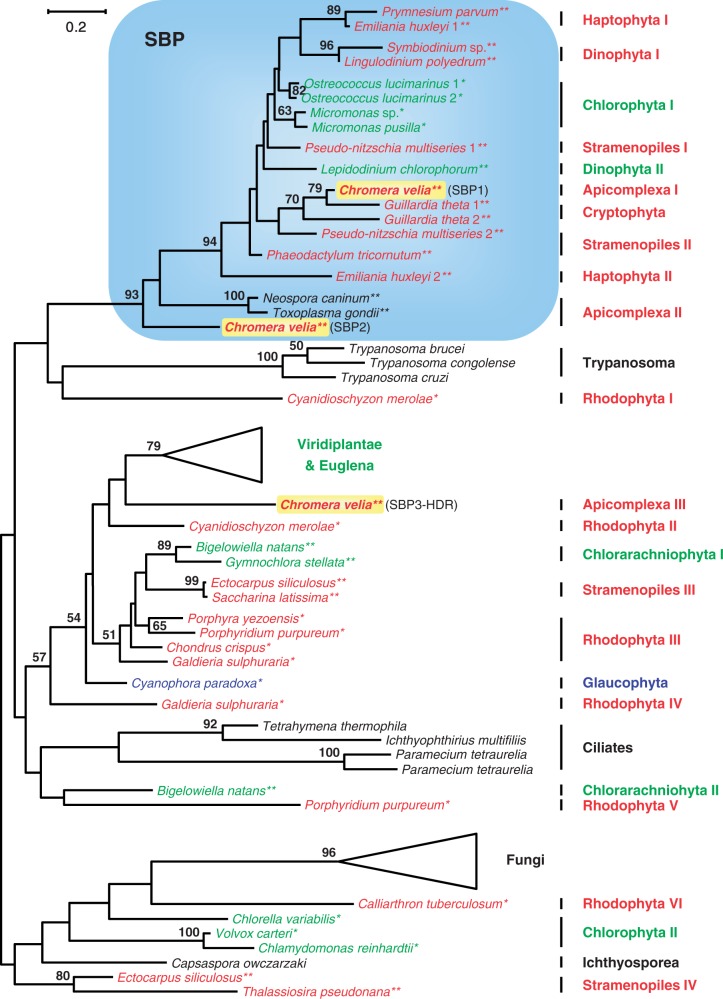
Phylogenetic ML RAxML analysis with a LG + F + Γ4 model of 80 SBP sequences based on 118 amino acid positions. The Calvin cycle-specific subtree of the CASH lineages is highlighted with a blue box. Distinct fungal and green plant subtrees have been merged; the complete phylogenetic tree is shown in supplementary figure S3, Supplementary Material online.

The SBP was the second “lucky marker” of the Calvin cycle that provided evidence for a common origin of red plastids from the CASH lineages (Teich et al. 2007). Though our new analysis recovered the corresponding subtree (blue box, fig. 3), it also revealed a previously unexpected complexity of SBP sequences. This complexity is exemplified by the presence of three distantly related homologs in *C. velia* and of at least two genes in some organisms belonging to several other lineages (e.g., Chlorophyta, Rhodophyta, stramenopiles, and Chlorarachniophyta). Additional genes may be indicative of a more versatile metabolism compared with those of land plants, in which a unique SBP is required for photosynthesis. Indeed, a nonhomologous SBP is essential for the NADPH-independent formation of ribose-5-phosphate in yeast (riboneogenesis; Clasquin et al. 2011), a function that could explain the presence of *sbp* genes in other nonphotosynthetic protists, such as trypanosomes, ciliates, and apicomplexan parasites (fig. 3; Teich et al. 2007).

All SBP sequences studied here can be traced back to an ancient duplication of the FBP (Martin et al. 1996). The common origin of photosynthetic SBPs from the CASH lineages, including SBP1 and SBP2 sequences from *Chromera* is evidenced by a long basal branch and a high statistical support (93% BP; blue box, fig. 3). However, the close relationship of the SBP1 from *Chromera* with sequences from the cryptophyte *Guillardia* is indicative of a HGT event. Moreover, the CASH subtree contains the only SBPs from Mamiellales (no additional isogenes; fig. S3*a*), thereby documenting a genuine case of gene replacement in these chlorophytes. Finally, the CASH cluster does not include all stramenopile sequences, which fall in at least three distant parts of the SBP tree. Such a patchy distribution may either be explained by HGTs or by the presence of several functional equivalent SBPs in the common stramenopile ancestor followed by differential gene loss due to metabolic reorganization in descending lineages. Although the abundance and functional flexibility of SBP genes hampers the development of a specific evolutionary scenario, the presence of the CASH subtree still supports the common origin of their plastids. Nevertheless, the deep nesting of *Chromera* SBP1 among *Guillardia* sequences (see earlier) and the basal position of its SBP2 close to the other apicomplexans to the exclusion of PCD homologs (94% BP, fig. 3) is incompatible with our null hypothesis of alveolate plastid monophyly.

The complexity of the SBP phylogeny is even exceeded by the phylogeny of the FBP, as reflected by the four genes in *C. velia* (supplementary table S1, Supplementary Material online). The plastid isoenzyme (FBPpla) is essential for the Calvin cycle, while the cytosolic equivalent (FBPcyt) is universally required for gluconeogenesis (Martin and Schnarrenberger 1997). With a focus on photosynthetic protists, we present here both a general overview of FBP sequences (supplementary fig. S3*b*, Supplementary Material online; compare with Teich et al. 2007) and a comprehensive analysis of the plastid isoenzymes (FBPpla; supplementary fig. S3*c*, Supplementary Material online). The distinct position of the three cytosolic copies of *Chromera* among bacterial FBPs is a noteworthy example of a transkingdom HGT in a nonendosymbiotic context (supplementary fig. S3*b*, Supplementary Material online). The xenologous gene replacement most likely occurred in a common ancestor of apicomplexans and *Perkinsus marinus*, a representative of the most basal lineage of dinoflagellates (Fernández Robledo et al. 2011). The absence of any orthologous sequences of the cytosolic FBP from PCDs, which are instead located in the eukaryotic part of the tree, might result from incomplete sequence sampling or be due to an independent replacement subsequent to the separation of Perkinsea. Moreover, the observation of two apicomplexan subtrees (supplementary fig. S3*b*, Supplementary Material online) reflects an early gene duplication that is suggestive of a complex metabolism or of a more versatile gene regulation in this alveolate lineage.

The topology of the plastid FBP sequences of the CASH lineages (blue box; supplementary fig. S3*c*, Supplementary Material online) is in agreement with a common red algal origin (see Teich et al. 2007), but the statistical support is low and the subtree contains the deeply nested FBPpla of *Euglena*. Here, the single FBApla of *Chromera* appears as an exception because many lineages, including plants, cryptophytes and chlorarachniophytes, actually contain two plastid genes. In contrast, the diatoms *Phaeodactylum* and *Pseudonitzschia* apparently exhibit four to six plastid isoenzymes, of which the respective functions are unknown. More generally, stramenopile sequences are distributed in seven separate branches (Stramenopiles I to VII, supplementary fig. S3*c*, Supplementary Material online), which rules out using this marker for deriving a scenario of the origin and evolution of this superensemble considered as a whole.

### Phylogenetic Analyses of Class I and Class II FBA Sequences

The presence of four class I and one class II FBA in *C. velia* documents the central function of this enzyme for the sugar phosphate metabolism. The FBA-I phylogeny shows a localization of *Chromera* sequences in four different subtrees, of which the evolutionary context is difficult to interpret (supplementary fig. S3*d*, Supplementary Material online). The maximally supported common basal branch of the subtree containing the FBA-Ic sequence from *C. velia* may reflect a specific metabolic role of the respective sequences. In fact, FBA-Ia is the only copy displaying the expected phylogenetic position, that is, basal to parasitic apicomplexan sequences (Apicomplexa III), and likely corresponds to the genuine cytosolic enzyme required for glycolysis. The observation of seven green plant subtrees (Viridiplantae I–VII) demonstrates the major roles of gene duplications and HGTs in FBA evolution. A phylogenetic pattern of similar complexity has been recently described in a study that focused on the subcellular localization of all five FBA enzymes of the diatom *Ph. tricornutum* (Allen et al. 2012). However, even complex phylogenies may feature specific groupings that can be used to draw conclusions on organismal evolution. One example is the distinct plastid subtree of class II FBAs (FbaC1), which contains cryptophyte, dinophyte (alveolate), stramenopile, and haptophyte sequences (see blue boxes, supplementary fig. S3*e*, Supplementary Material online), and has provided evidence for the common origin of CASH plastids (fourth lucky gene; Patron et al. 2004). In contrast, our analyses of the complete draft genome of *Chromera* reveals that this alga lacks the CASH-specific plastid class II aldolase, even if we have identified a more distantly related cytosolic isoenzyme (supplementary fig. S3*e*, Supplementary Material online). The absence of the plastid *fba*C1 gene may result from a lineage-specific functional replacement followed by the loss of the original gene or it reflects the recruitment of a plastid aldolase in *C. velia* subsequent to an independent eukaryote-to-eukaryote endosymbiosis.

### Phylogenetic Analyses of GAPDH Sequences

**Fig. 4.—:**
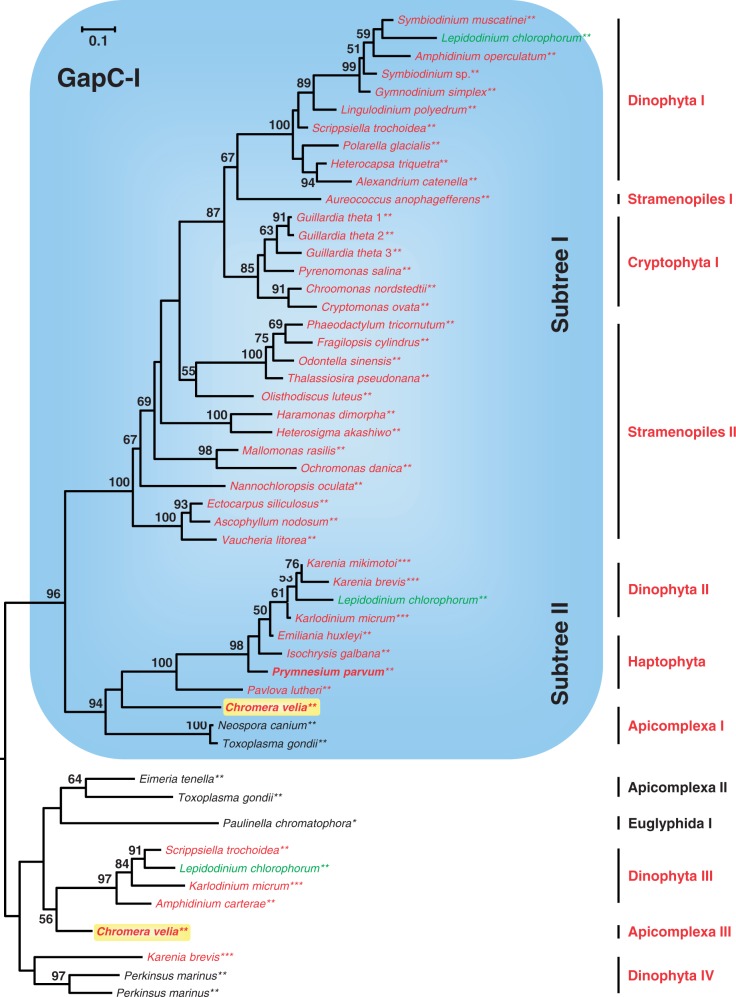
Image detail of a phylogenetic ML analysis of GAPDH sequences rooted on the cytosolic GapC sequences from alveolates. The blue box highlights the bipartite subtree of plastid GapC-I sequences of CASH lineages, which are essential for the Calvin cycle. The complete RAxML analysis was performed with a LG + F + Γ4 model of 95 GAPDH sequences based on 194 amino acid positions (see supplementary fig. S8, Supplementary Material online). Species names are shown in red and green according to the rhodophycean or chlorophycean origin of their complex plastids, and species with heterotrophic plastids are shown in black. The number of asterisks at the species names indicates the current state of knowledge about the origin of their plastids via primary (*), secondary (**), or tertiary (***) endosymbiosis.

The characteristic plastid GAPDH of the CASH lineages, known as GapC-I, is also present in *Chromera* (fig. 4; supplementary fig. S4, Supplementary Material online; Oborník et al. 2009). This enzyme is a duplicate of the cytosolic GapC that obtained a bipartite signal-/transit-peptide for plastid import and adapted its cosubstrate specificity from NAD^+^ to NADPH to fulfill its novel function in the anabolic Calvin cycle (Clermont et al. 1993). The GapC-I replaced the original red algal GapA of cyanobacterial origin in the course of the secondary endosymbiosis (Liaud et al. 1997) and, as such, is a prime example of endosymbiotic gene replacement. Because it is very unlikely that all lineages of complex algae with red plastids (CASH; fig. 4) lost their *gapA* gene and recruited their *gapC*-I gene independently, the observed distribution provides a strong evidence for a common origin of CASH plastids, including those of dinoflagellates, *Chromera,* and apicomplexan parasites. Consequently, the designation lucky gene remains justified, even with the expanded species sampling of the present study. The unique cosubstrate adaptation of the GapC-I enzyme correlates with the long basal branch of the CASH sequences, and its new anabolic function likely explains the absence of other isoenzymes.

Obviously, the GapC-I tree (fig. 4) is not compatible with a vertical evolution of CASH plastids because accepted host-cell relationships are not recovered (e.g., stramenopiles and alveolates). However, the observed distribution could either fit a scenario postulating higher-order endosymbioses or the default hypothesis of several random recruitments via independent HGTs. The phylogeny of plastid GapC-I sequences is deeply divided into two well-supported subtrees containing stramenopile, cryptophyte, and dinophyte sequences on the one side versus apicomplexan and haptophyte sequences on the other side. Taken at face value, the paraphyly of stramenopiles in subtree I suggests that their genes may have been transmitted to dinoflagellates and cryptophytes (fig. 4). The observation of dinophyte sequences among haptophytes in subtree II is the consequence of a well-known higher-order endosymbiosis involving a representative of Prymnesiales (Gabrielsen et al. 2011). However, the haptophyte origin of the second GapC-I sequence from the green dinoflagellate *L. chlorophorum*, which obtained its current plastid by a secondary endosymbiosis with a chlorophyte (Matsumoto et al. 2011), is more difficult to explain, especially considering the persistence of the original gene (in subtree I) in this huge genome (Minge et al. 2010). The a priori surprising presence of a plastid GAPDH in the apicomplexan parasites *Toxoplasma* and *Neospora* argues for an additional function, apart from its canonical role within the Calvin cycle (see also Lizundia et al. 2009), though its close relationship to the *Chromera* gene is compatible with a common photosynthetic ancestry. In contrast, the distinct localization of GapC-I sequences from Apicomplexa (subtree II) and PCDs (subtree I; fig. 4) strongly supports an independent origin of this gene in the two plastid-containing lineages of alveolates, thus contradicting again our null hypothesis.

### Phylogenetic Analyses of HDR Sequences

**Fig. 5.—:**
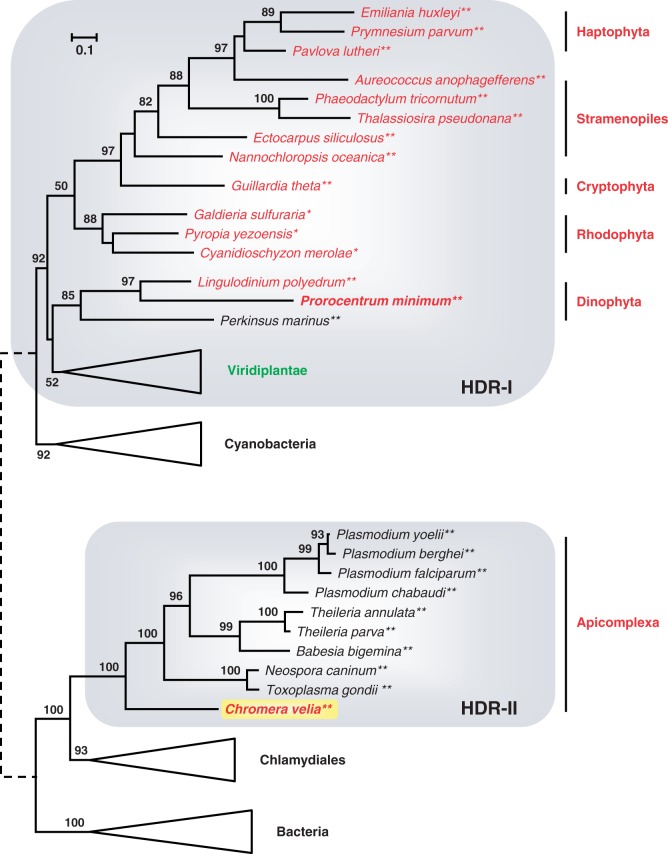
Merged phylogenetic ML tree of HDR sequences of plastid-specific isoprenoid biosynthesis (MEP-pathway). The RAxML analyses of HDR subtrees I and II were performed with a LG + F + Γ4 model of 40 and 45 HDR sequences based on 288 and 257 amino acid positions, respectively (see supplementary fig. S9*a* and *b*, Supplementary Material online). The gray boxes indicate eukaryotic branches of the subtrees.

The HDR of the SBP3-HDR fusion protein from *C. velia* (fig. 1) was analyzed as a sixth marker of the primary metabolism. HDR enzymes catalyze the final step in the plastid-specific 2-C-methyl-d-erythritol 4-phosphate (MEP) pathway of isoprenoid biosynthesis (Grauvogel and Petersen 2007). The presence of a single *hdr* gene in the draft genome of *C. velia* (see earlier) is typical for plastid-containing eukaryotes and in agreement with its monospecific function. HDR sequences from several apicomplexan parasites and the dinophyte oyster pathogen *Pe. marinus* were retrieved from public databases. However, the expression level of MEP genes is generally lower compared with those of the Calvin cycle and the number of available HDR sequences is consequently limited. To compare HDRs from the two lineages of photosynthetic alveolates (PCDs and Apicomplexa), we established a full-length cDNA from the PCD *P. minimum*. Our initial phylogenetic HDR analyses revealed two discrete eukaryotic subtrees within the bacterial diversity (data not shown). In order to maximize phylogenetic resolution, we separately analyzed these two eukaryotic subtrees, along with their closest bacterial outgroups (supplementary fig. S5*a* and *b*, Supplementary Material online) and present the composite tree in figure 5. The cyanobacterial roots of subtree I, which contains all plastidial eukaryotes except Apicomplexa, indicate that the *hdr* gene was acquired by EGT during the establishment of the primary plastid. Moreover, assuming that the position of dinophytes is due to an LBA artifact, the topology supports the red algal origin of plastid genes in CASH lineages. Interestingly, all apicomplexan HDR sequences are part of the distantly related subtree II and the basal branching of *Chromera* is in agreement with a photosynthetic ancestry of the whole lineage (fig. 5). Because neither the draft genome of *Pe. marinus* nor our transcriptome of *P. minimum* (data not shown) did yield an additional dinophyte copy belonging to subtree II, the HDR phylogeny is also incompatible with the null hypothesis of a common endosymbiotic origin of apicomplexan and PCD plastids.

### Phylogenetic Analyses of the Plastid Import Machinery (Der1, Cdc48, Tic20, Tic110)

**Fig. 6.—:**
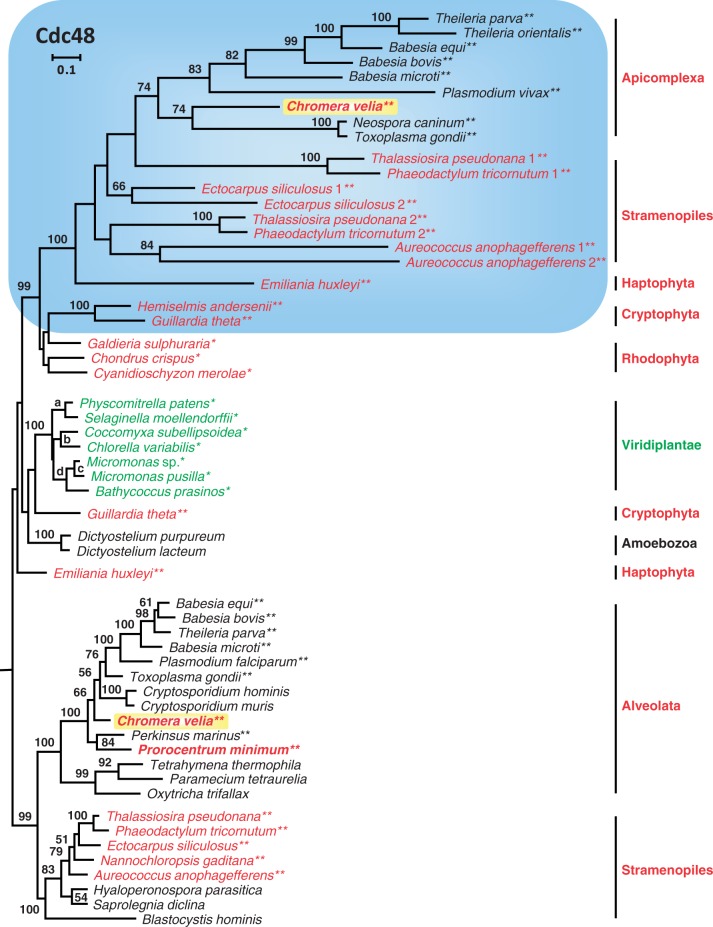
Phylogenetic ML RAxML analysis with a LG + F + Γ4 model of 68 Cdc48 sequences of the ER-associated degradation (ERAD) system based on 618 amino acid positions. The subtree of the SELMA for protein transport through the periplastidal plastid membrane of CASH lineages is highlighted with a blue box. The complete phylogenetic tree is shown in supplementary fig. S6*b*, Supplementary Material online.

In addition to the six genes of the primary metabolism, we analyzed four essential markers of the protein import machinery of plastids in CASH lineages (Stork et al. 2013). Sixteen novel cDNA sequences of *Chromera* and *Prorocentrum* have been deposited in GenBank (KJ194480–KJ194495). First, we analyzed derlin family proteins, which are required for both the ER-associated protein degradation system (ERAD) and the symbiont-specific ERAD-like machinery (SELMA) for transport through the periplastidal membrane of complex red plastids (supplementary fig. S6*a*, Supplementary Material online; Sommer et al. 2007). Considering the limited number of positions used for the Der1 phylogeny (106 amino acids), the observed statistical support is actually strong for several branches. There are three distinct alveolate subtrees, each of them containing a single *Chromera* sequence. In contrast, *P. minimum* and *Pe. marinus* sequences are only found in the two ERAD subtrees corresponding to the host-cell protein degradation machinery. The two latter subtrees also contain sequences of the aplastidial apicomplexan parasite *Cryptosporidium* (Huang et al. 2004). The essentiality of the plastid-specific Der1 protein has been documented for *Toxoplasma gondii* (Agrawal et al. 2009). Thus, its absence in dinoflagellates is likely indicative for differences in the protein import mechanism. Second, the analysis of Cdc48, an ATPase of which the cytosolic form has been described as “a power machine in protein degradation” (Stolz et al. 2011), exhibits a comparable picture. Although the cytosolic Cdc48 subtree of alveolates (represented by ciliates, dinoflagellates, and apicomplexans) reflects the host-cell branching pattern (fig. 6), plastid equivalents are lacking in dinophycean transcriptomes, including those of *P. minimum*, and in the genome of *Pe. marinus*. Along with Der1, the plastid form of Cdc48 is an essential component of protein transport through the periplastidal membrane of apicomplexans (Agrawal et al. 2009) and is present in *Chromera* and in all CASH lineages with plastids surrounded by four membranes. The highly supported common red algal origin of the plastid Cdc48 is highlighted by the blue box in figure 6. This plastid marker is a perfect example of EGT because it is nucleomorph-encoded in cryptophytes (Douglas et al. 2001; Lane et al. 2007), whereas long branches of apicomplexans, stramenopiles, and haptophytes correlate with its successful transfer into the nucleus of the respective host cells. The nested and well-supported localization of the plastid Cdc48 of *Chromera* within apicomplexan parasites supports a common origin of their plastids. In contrast, the absence of both plastid Cdc48 and Der1 genes in dinoflagellates suggests that protein import through the outermost membrane of current PCD plastids is organized differently.

Finally, we also analyzed crucial components of protein trafficking through the innermost membrane of all plastids. Hence, Tic20 was chosen as a third marker due to its essential role in plastid protein import, including the apicoplast of *T. gondii* (van Dooren et al. 2008). Our phylogenetic analysis reveals the expected localization of *Chromera* at the basis of plastid-containing apicomplexans, whereas all five expressed *Prorocentrum* sequences group together with stramenopiles (supplementary fig. S6*b*, Supplementary Material online). The presence of multiple copies of important genes has previously been described for PCDs and reflects their peculiar genome organization (Lin 2011). Tic20 is of cyanobacterial origin but further conclusions about the evolutionary relationships among CASH lineages cannot be drawn due to the limited number of unambiguously aligned amino acid positions. Instead, we turned to the large Tic110 gene (1174 amino acid positions in *Chromera*) as our fourth marker. Unfortunately, Tic110 sequences exhibit a very low degree of conservation, which left us with only 121 amino acid positions for phylogenetic analysis. Even though poorly resolved, the resulting tree (supplementary fig. S6*c*, Supplementary Material online) contains representatives of all CASH lineages, among which the unique transcript of *Prorocentrum* is robustly located in a stramenopile subtree (86% BP) to the exclusion of *Chromera*. Therefore, in agreement with Der1 and Cdc48, none of the two Tic markers support a common origin of *Chromera* and PCDs, which again contradicts the null hypothesis of shared plastid ancestry.

## Discussion

### High-Quality Transcripts and the Draft Genome of *C. velia*

In the present work, we used the Illumina technology to establish both a specific set of cDNAs and a draft meta-genome of *C. velia*, which represents a key species for the study of plastid evolution. Next-generation sequencing allowed us to generate a very large amount of data and to reach a sufficient sequencing depth, as evidenced by the fact that all novel TSAs represent full-length protein sequences (supplementary table S1, Supplementary Material online). The exceptional quality of our cDNAs is reflected by very large de novo assembled TSAs (>13 kb). Moreover, we sequenced the genome of *C. velia* at 18-fold coverage (Accession: ARZB00000000) and assembled a draft version of the plastome (see Janouskovec et al. 2010) as well as a tiny mitochondrial genome, only 2 kb in size and bearing the *coxI* gene (supplementary fig. S1, Supplementary Material online). To our knowledge, the latter is the smallest organelle genome identified so far, and it likely represents the last stage before the complete loss of the endosymbiotic dowry observed in anaerobic hydrogenosomes (Palmer 1997). Finally, with respect to the analysis of the five Calvin cycle markers, our combined sequencing approach ensured the identification of a total of 15 homologous genes and allowed us to assert that these are all expressed and of eukaryotic origin (supplementary table S1, Supplementary Material online).

### Lucky Genes and Evolution of CASH Plastids—What Is Left?

Building on our high-quality sequence data from *C. velia* (supplementary table S1, Supplementary Material online), we presented a phylogenetic update of the five Calvin cycle markers (PRK, SBP, FBP, FBA, GAPDH) that support a common origin of CASH plastids (Harper and Keeling 2003; Patron et al. 2004; Petersen et al. 2006; Teich et al. 2007). In particular, our expanded trees allowed us 1) to critically reevaluate the actual evidence for a common ancestry of CASH plastids and 2) to test the prediction of a common ancestry of alveolate plastids first suggested by the now defunct chromalveolate hypothesis (Cavalier-Smith 1999; Baurain et al. 2010). For the first point, our phylogenetic analyses of the PRK, SBP, FBP, and GAPDH genes (figs. 2–4, supplementary fig. S3*c*, Supplementary Material online) confirm the existence of CASH-specific plastid subtrees also containing sequences from *Chromera* (highlighted by blue boxes). This is in agreement with former studies based on smaller data sets (Harper and Keeling 2003; Petersen et al. 2006; Teich et al. 2007). The only exception is the plastid class II aldolase (Patron et al. 2004), which is missing from the genome of *C. velia* (supplementary table S1 and fig. S3*e*, Supplementary Material online) and of which the metabolic function in the Calvin cycle is probably maintained by a nonhomologous class I isoenzyme (supplementary fig. S3*d*, Supplementary Material online). Nevertheless, four lucky genes still support a common plastid ancestry of cryptophytes, alveolates (PCDs and Apicomplexa), stramenopiles, and haptophytes via a single secondary endosymbiosis with a red alga. Concerning the second point, the falsification of the chromalveolate hypothesis has resulted in the realization that higher-order endosymbioses might be more common than previously assumed (Archibald 2009; Baurain et al. 2010). Therefore, many of the former interpretations about plastid evolution are in need of a reexamination. One example is the proposed common endosymbiotic origin of alveolate plastids that is supported by the presence of a proteobacterial type II RuBisCo in PCDs and *Chromera* (Janouskovec et al. 2010) and was used here as a testable null hypothesis. All six nuclear-encoded plastid specific genes of primary metabolism considered in the present study (PRK, SBP, FBPpla, FBA-II, GapC-I, HDR) were individually integrated into the host-cell nucleus after eukaryote-to-eukaryote endosymbioses; yet, not a single marker supports the common ancestry of alveolate plastids. Indeed, while the relationships are not resolved in PRK and plastid FBP phylogenies (fig. 2, supplementary fig. S3*c*, Supplementary Material online), the plastid SBP subtree documents the basal position of apicomplexans including *Chromera* (SBP2) to the exclusion of PCDs (fig. 3). Moreover, *Chromera* does not contain the plastid class II aldolase typical of the CASH lineages (supplementary fig. S3*e* and table S1, Supplementary Material online), whereas the plastid GAPDH exhibits a deep split between PCDs and apicomplexans (fig. 4). In the latter case, it is unlikely that the two GapC-I subtrees stem from an early gene duplication followed by independent differential losses, as previously proposed (Takishita et al. 2009), because such losses should have occurred in five lineages in parallel (cryptophytes, haptophytes, stramenopile algae, and the two lineages of photosynthetic alveolates). Finally, the characteristic apicomplexan HDR is a chlamydial xenolog that replaced the typical eukaryotic gene (fig. 5). In our opinion, the lack of a unifying support for a common endosymbiotic origin of alveolate plastids is not due to phylogenetic artifacts, such as the LBA that obscured the Mamiellales origin of the green PRK in CASH plastids (fig. 2, supplementary fig. S2, Supplementary Material online). Instead, we argue that the apparent conflicts between different plastid markers result from individual HGT events, especially if *Chromera* and/or PCD sequences robustly branch with other organisms in the tree. Because all six markers are unlikely to have been systematically replaced in one of the two lineages, we conclude that the current plastids from Apicomplexa (represented by *Chromera*) and PCDs likely originated in two independent endosymbiotic events.

This conclusion is in agreement with the fundamental differences of the plastome architecture (Zhang et al. 1999; Janouskovec et al. 2010) and the morphological difference of four versus three plastid membranes in *Chromera* and PCDs, respectively (Graham and Wilcox 2000; Moore et al. 2008). Remarkably, the deviating plastid ultrastructure correlates with a different mechanism of protein import in PCDs compared with closely related apicomplexans (Nassoury et al. 2003). This finding is supported by our analyses of components of the plastid import system focused on *Chromera* and *Prorocentrum* (fig. 6, supplementary fig. S6, Supplementary Material online). The symbiont-specific ERAD-like machinery (SELMA), which is required for the transfer of proteins through the periplastidal membrane, is present in all CASH lineages with plastids surrounded by four membranes (Stork et al. 2013), thus providing independent evidence for a common origin of their complex plastids. Indeed, it is generally assumed that this sophisticated, multiprotein machinery has not originated several times independently. Further support for a shared ancestry is provided by the functional conservation of bipartite signal-transit peptide recognition motifs that ensure a correct protein trafficking into complex plastids (Kilian and Kroth 2005; Gruber et al. 2007). The SELMA apparatus has evolved by functional recycling of preexisting components after secondary endosymbiosis (Bolte et al. 2011). Hence, the essential ATPase Cdc48 (Agrawal et al. 2009), which is still nucleomorph-encoded in cryptophytes (Douglas et al. 2001; Lane et al. 2007), reflects a genuine EGT from the engulfed red alga (fig. 6). However, the notable absence of plastid Der1 and Cdc48 genes in dinoflagellates contrasts with the ubiquity of SELMA in other CASH lineages (fig. 6, supplementary fig. S6*a* and *b*, Supplementary Material online) and suggests that PCDs use a different import system for crossing the outermost plastid membrane (Nassoury et al. 2003). In contrast, the presence of Tic20 and Tic110 in *Chromera* and *Prorocentrum* confirms the universal conservation of the basic transport machinery through the innermost plastid membrane, but our phylogenetic analyses indicate that these genes were independently established in the nuclear genome of the two lineages of plastid-containing alveolates (supplementary fig. S6*c* and *d*, Supplementary Material online). Accordingly, SELMA and Tic markers all provide additional support for our conclusion that the morphologically different plastids of Apicomplexa and PCDs likely originated in two independent higher-order endosymbioses.

### Lucky Genes and the Evolution of CASH Plastids—What Is Next?

In spite of the body of evidence discussed above, our results are at odds with the known distribution of RuBisCo genes. Indeed, the nuclear-encoded type II RuBisCo has exclusively been reported in PCDs and photosynthetic apicomplexans (Morse et al. 1995; Janouskovec et al. 2010). Even if RuBisCo sometimes yield puzzling phylogenies (see, e.g., Delwiche and Palmer 1996) and albeit it may result from HGT in a nonendosymbiotic context (default hypothesis that is difficult to put to test), we agree that this observation needs to be explained. To this end, we see at least three possibilities. First, the most parsimonious scenario (with respect to the number of required endosymbioses) posits the recruitment of the complex plastid in a common ancestor of apicomplexans and dinoflagellates. In its standard formulation, it implies the loss of one plastid membrane in PCDs concomitant with a substantial reorganization of their plastid import system. In light of our phylogenies, we are not convinced that this hypothesis, even if commonly taken as granted, is the best one, because it would entail many HGTs to completely erase the historical signal from the ten markers analyzed here. The second scenario also assumes a common plastid origin of both alveolate lineages but then proposes a subsequent endosymbiotic replacement of the original complex plastid in PCDs from an undetermined donor, for example, a haptophyte (Shalchian-Tabrizi et al. 2006) or a stramenopile (supplementary fig. S6*c* and *d*, Supplementary Material online). Accordingly, the plastid of *Perkinsus*, which represents the most basal dinoflagellate lineage and likely contains four membranes (Grauvogel et al. 2007; Teles-Grilo et al. 2007), would still reflect the ancient status. Third, the largely different plastid morphology of PCDs may be the result of the engulfment and subsequent reduction of an apicomplexan alga. This scenario would also explain shared traits among plastid-containing alveolates, like the 23S rDNA, type II RuBisCo and polyuridylylation of plastid gene transcripts (Zhang et al. 2000; Wang and Morse 2006; Janouskovec et al. 2010), which is missing in *Plasmodium* (Dorrell et al. 2014). Hence, the observed incongruent phylogenies would simply reflect stochastic gene recruitments in the context of independent higher-order endosymbioses. The third scenario also implies that the absence of chlorophyll *c* in chromerids (Moore et al. 2008; Oborník et al. 2012) is the consequence of secondary loss.

Future analyses should thus aim at distinguishing between these three possibilities. However, developing a compelling scenario of plastid endosymbioses in alveolates is only the first step toward understanding plastid evolution in all CASH lineages. With respect to prospective studies, phylogenomic analyses of plastome and host-cell data sets should serve as references, but nuclear-encoded plastid markers comparable to our lucky genes are those that offer the most promising perspectives for resolving the endosymbiotic puzzle. Their diagnostic power stems from the relaxed selective pressure immediately following gene transfer (HGT, EGT) and the establishment in the host-cell nucleus, but the crucial question is which of them are also good markers to decrypt ancient CASH-related endosymbioses? Our phylogenetic analyses of enzymes such as SBP, FBP, FBA-I, and FBA-II (see supplementary figs. S3*a–e*, Supplementary Material online), which are simultaneously involved in different metabolic pathways (Calvin cycle, glycolysis, gluconeogenesis) revealed frequent gene duplications, HGTs, and examples of functional gene replacements. The same is true for the markers of the SELMA machinery for protein import into complex plastids that have been recycled from a preexisting translocation system (Bolte et al. 2011). Because most of these markers are not suited to unambiguously retrace endosymbiotic events, future studies should focus on single-copy genes in order to overcome these problems. Nuclear-encoded enzymes of the plastid MEP-pathway for isoprenoid biosynthesis, such as the HDR (fig. 5), and plastid-specific enzymes for fatty acid biosynthesis are auspicious markers for unraveling the endosymbiotic origins of complex algae (Ralph et al. 2004; Frommolt et al. 2008; Lizundia et al. 2009). Confounding isoenzymes are inexistent and their nonphotosynthetic plastid function opens the perspective to include heterotrophic protists like the malaria parasite into the phylogenies. Finally, comparative analysis of multiple data sets should allow distinguishing between sporadic HGT and authentic EGT.

### The Rhodoplex Hypothesis

The last decade of research in plastid evolution has been dominated by the dispute over Cavalier-Smith’s (1999) “chromalveolate hypothesis.” His parsimony-based scenario proposing that “chromists” (haptophytes, cryptophytes, stramenopiles) and alveolates (dinoflagellates, apicomplexans, ciliates) originated from a single secondary endosymbiosis with a rhodophyte was bright and highly stimulating but eventually revealed to be incorrect (Baurain et al. 2010). Meanwhile, former proponents of the concept have even begun to bring evidence for the separate origins of the host cells of some lineages, such as haptophytes and cryptophytes (Burki et al. 2012). However, a major taxonomic burden of the past is the (meanwhile corrected) classification scheme of Adl et al. (2005, 2012), who ennobled the chromalveolates to a new eukaryotic superensemble. For a while, the underlying hypothesis was close to become a self-fulfilling prophecy, as many plastid-related articles of the time had to include the comical precaution “If the chromalveolate hypothesis is correct …” (Bachvaroff et al. 2005; Okamoto and Inouye 2005; Gould et al. 2008; Oborník et al. 2009). In some cases, enthusiastic adoption of this hypothesis resulted in wrong conclusions being drawn from otherwise valid data (compare, e.g., Matsuzaki et al. [2008] with Grauvogel et al. [2007]). Worse, in recent review articles about plastid evolution, the term chromalveolates is still in use as it was a valid taxonomic unit (Green 2011; Dorrell and Smith 2011). Therefore, for the sake of avoiding further confusion in the future, we recommend to discard this term and instead use the operational phrase “CASH lineages” (Baurain et al. 2010) to agnostically designate the independent groups of “complex algae with red plastids” (Petersen et al. 2006).

**Fig. 7.—:**
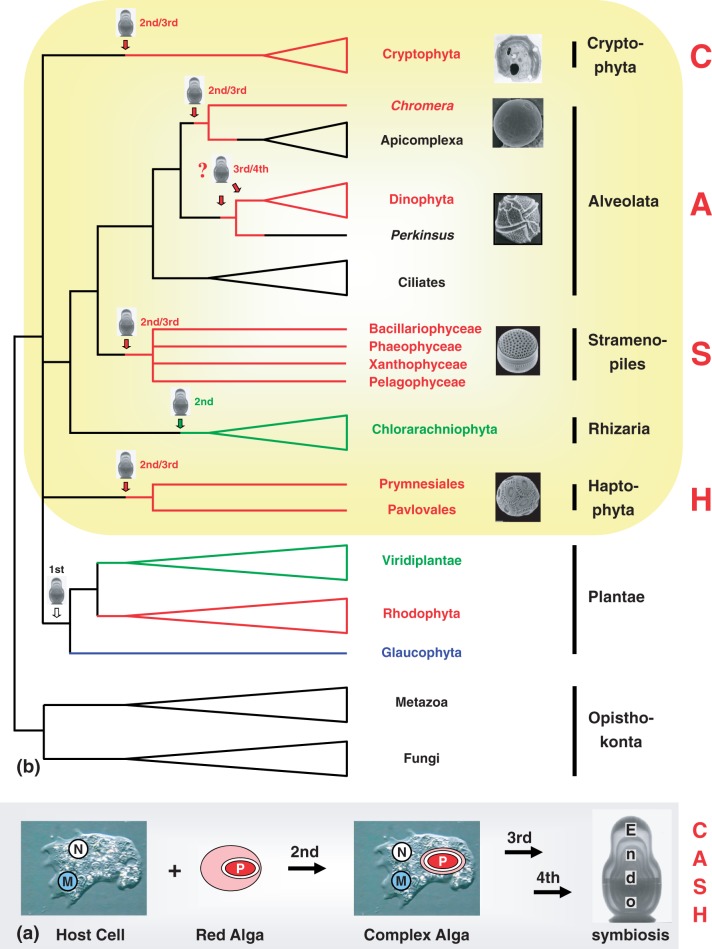
(*a*) Origin of complex algae with red plastids via a single secondary endosymbiosis with a red alga and successive tertiary and quaternary endosymbioses. N: nucleus; M: mitochondrion; P: plastid. (*b*) Scenario of plastid evolution among CASH lineages according to the rhodoplex hypothesis. X-ray images of the Russian Matryoshka dolls indicate independent events of plastid endosymbioses. All CASH plastids originate from an initial engulfment of a rhodophyte (see [*a*]), but the genuine secondary endosymbiont and the order of subsequent endosymbioses remains to be determined (indicated by 2nd/3rd and 3rd/4th). The typical plastid of PCD may represent a reduced apicomplexan alga (see current study). The gain of rhodophycean plastids as well as the loss of photosynthesis/plastids is indicated by the red horizontal lines. With respect to stramenopiles, only a subset of separate lineages is shown. Micrograph courtesy of Peter Vontobel, Sven Gould, Woody Hastings, and Manfred Rohde.

In this postchromalveolate era, there is room for a hypothesis that could make sense of all the available evidence while opening new avenues for research in plastid evolution. The main tenets of this alternative scenario are known: 1) the complex plastids from CASH originate from a single secondary endosymbiosis with a rhodophyte and 2) subsequent eukaryote-to-eukaryote endosymbioses are needed to explain the incongruence observed between plastid-encoded and nuclear-encoded markers (Teich et al. 2007; Baurain et al. 2010). We propose to name it the rhodoplex hypothesis (fig. 7). It is not as arbitrary as the portable plastid hypothesis (Grzebyk et al. 2003) or as the idea of serial endosymbioses (Dorrell and Smith 2011), because it explicitly retains the initial secondary endosymbiosis with a red alga as the first step that eventually resulted in the extant diversity of the complex red lineages (illustrated by Delwiche 1999). The rhodoplex hypothesis is compatible with the main conclusions of several recent studies (see, e.g., Bachvaroff et al. 2005; Bodył and Moszczynski 2006; Teich et al. 2007; Bodył et al. 2009; Woehle et al. 2011). Moreover, it may be extended by an additional ancestral cryptic endosymbiosis, were it needed for explaining the apparent excess of xenologous genes from distinct sources found in the nuclear genomes of CASH lineages (Huang and Gogarten 2007; Moustafa et al. 2009; but see also Woehle et al. 2011; Deschamps and Moreira 2012). Finally, it should serve as an impulsion for the successful development of a comprehensive scenario for the evolution of complex red plastids.

## Supplementary Material

Supplementary figures S1–S6 and table S1 are available at *Genome Biology and Evolution* online (http://www.gbe.oxfordjournals.org/).

Supplementary Data
