# Supplementary material for: Chromera velia, Endosymbioses and the Rhodoplex Hypothesis—Plastid Evolution in Cryptophytes, Alveolates, Stramenopiles, and Haptophytes (CASH Lineages)
Source: Genome Biol Evol. 2014 Feb 25;6(3):666–84. doi: 10.1093/gbe/evu043 (PMC3971594; doi:10.1093/gbe/evu043)
Supplement: Supplementary Data [file supp_6_3_666__index.html]

Chromera velia, Endosymbioses and the Rhodoplex Hypothesis - Plastid Evolution in Cryptophytes, Alveolates, Stramenopiles and Haptophytes (CASH Lineages) — Chromera velia, Endosymbioses and the Rhodoplex Hypothesis—Plastid Evolution in Cryptophytes, Alveolates, Stramenopiles, and Haptophytes (CASH Lineages) — Supplementary Data 

# *Chromera velia*, Endosymbioses and the Rhodoplex Hypothesis—Plastid Evolution in Cryptophytes, Alveolates, Stramenopiles, and Haptophytes (CASH Lineages)

## Supplementary Data

files

**Files in this Data Supplement:**

- Supplementary Data - pdf file
- Supplementary Data - pdf file
- Supplementary Data - pdf file
- Supplementary Data - pdf file
- Supplementary Data - pdf file
- Supplementary Data - pdf file
- Supplementary Data - pdf file
