# Supplementary material for: Chromera velia, Endosymbioses and the Rhodoplex Hypothesis—Plastid Evolution in Cryptophytes, Alveolates, Stramenopiles, and Haptophytes (CASH Lineages)
Source: Genome Biol Evol. 2014 Feb 25;6(3):666–84. doi: 10.1093/gbe/evu043 (PMC3971594; doi:10.1093/gbe/evu043)
Supplement: Supplementary Data [file supp_evu043_Petersen_FigS1.pdf]

Mitochondrial genome - *Chromera velia* CCAP 1602/1 (KC899110)

```
                 ....|....| ....|....| ....|....| ....|....| ....|....| ....|....| ....|....| ....|....| ....|....|
                         10         20         30         40         50         60         70         80         90
Consensus        ---------- ---------- ---------- ---------- ---------- ---------- ---------- ---------- ----------
CoxI_PCR         ---------- ---------- ---------- ---------- ---------- ---------- ---------- ---------- ----------
Illumina_1       ---------- ---------- ---------- ---------- ---------- ---------- ---------- ---------- ----------
Illumina_2       ---------- ---------- ---------- ---------- ---------- ---------- ---------- ---------- ----------
Illumina_3       ---------- ---------- ---------- ---------- ---------- ---------- ---------- ---------- ----------
Illumina_4       ---------- ---------- ---------- ---------- ---------- ---------- ---------- ---------- ----------
Illumina_5       ---------- ---------- ---------- ---------- ---------- ---------- ---------- ---------- ----------
iPCR_1 (5')      ---------- -------GAT TTGGTAAGTA AGAGGAATGC CTGTATGGGA CAGTCGTATG CCGGGTTATA ACAGACCAAC AACACCTTTC
iPCR_2 (5')      TTGCTGGGTT AGCTAGTGAT TTGGTATGTA AGAGGAATGC CTGTATGGGA CAGTCGTATG CCGGGTTATA ACAGACCAAC AACACCTT-C
iPCR_3 (5')      TTGCTGGGTT AGCTAGTGAT TTGGTATGTA AGAGGAATGC CTGTATGGGA CAGTCGTATG CCGGGTTATA ACAGACCAAC AACACCTT-C
iPCR_4 (5')      ---------- ---------- ---------- ---------- ---------- ---------- ---------- ---------- -------TTG
iPCR_4 (3')      ---------- ---------- ---------- ---------- ---------- ---------- ---------- ---------- ----------
iPCR_3 (3')      ---------- ---------- ---------- ---------- ---------- ---------- ---------- ---------- ----------
iPCR_2 (3')      ---------- ---------- ---------- ---------- ---------- ---------- ---------- ---------- ----------
```

```
                 ....|....| ....|....| ....|....| ....|....| ....|....| ....|....| ....|....| ....|....| ....|....|
                        100        110        120        130        140        150        160        170        180
Consensus        ---------- ---------- ---------- ---------- ---------- ---------- ---------- ---------- ----------
CoxI_PCR         ---------- ---------- ---------- ---------- ---------- ---------- ---------- ---------- ----------
Illumina_1       ---------- ---------- ---------- ---------- ---------- ---------- ---------- ---------- ----------
Illumina_2       ---------- ---------- ---------- ---------- ---------- ---------- ---------- ---------- ----------
Illumina_3       ---------- ---------- ---------- ---------- ---------- ---------- ---------- ---------- ----------
Illumina_4       ---------- ---------- ---------- ---------- ---------- ---------- ---------- ---------- ----------
Illumina_5       ---------- ---------- ---------- ---------- ---------- ---------- ---------- ---------- ----------
iPCR_1 (5')      TGTTCATCCC TCTATAGCTA TATGTACTGT GTGTGTGGGA TTATTGGTAG AACACGGTTA TTCATTATCT ATGCCCACAT GCCTGTGGGA
iPCR_2 (5')      TGTTCATCCC TCTATAGCTA TATGTACTGT GTGTGTGGGA TTATTGGTAG AACACGGTTA TTCATTATCT ATGCCCACAT GCCTGTGGGA
iPCR_3 (5')      TGTTCATCCC TCTATAGCTA TATGTACTGT GTGTGTGGGA TTATTGGTAG AACACGGTTA TTCATTATCT ATGCCCACAT GCCTGTGGGA
iPCR_4 (5')      CTGGGTTAGC TAGTGATTTG GTATGTAAGA GGAATGCCTG TATGGGACAG TCGTATGCCG GGTTATAACA GACCAACAGC ACCTTCTGTT
iPCR_4 (3')      ---------- ---------- ---------- ---------- ---------- ---------- ---------- ---------- ----------
iPCR_3 (3')      ---------- ---------- ---------- ---------- ---------- ---------- ---------- ---------- ----------
iPCR_2 (3')      ---------- ---------- ---------- ---------- ---------- ---------- ---------- ---------- ----------
```

Figure S1

Mitochondrial genome - *Chromera velia* CCAP 1602/1 (KC899110)

	190	200	210	220	230	240	250	260	270
**Consensus**	----------	----------	----------	----------	----------	----------	----------	----------	**TAGTTCAGAT**
**CoxI_PCR**	----------	----------	----------	----------	----------	----------	----------	----------	----------
**Illumina_1**	----------	----------	-------TGT	GTGGGTAAGG	TTGGACAAGG	ACTCCTGCAT	TATATTGTGT	GCCTCATCGC	TAGTTCAGAT
**Illumina_2**	----------	----------	----------	----------	----------	----------	----------	----------	----------
**Illumina_3**	----------	----------	----------	----------	----------	----------	----------	----------	----------
**Illumina_4**	----------	----------	----------	----------	----------	----------	----------	----------	----------
**Illumina_5**	----------	----------	----------	----------	----------	----------	----------	----------	----------
**iPCR_1 (5′)**	GTGTTGGATA	AATATTAGAG	TTGAACACAC	ACAGATAACA	CACACAGATA	ATATTAATAG	AACGGTTATA	CGTGGGTTGG	TAGTTCAGAT
**iPCR_2 (5′)**	GTGTTGGATA	AATATTAGAG	TTGAACACAC	ACAGATAACA	CACACAGATA	ATATTAATAG	AACGGTTATA	CGTGGGTTGG	TAGTTCAGAT
**iPCR_3 (5′)**	GTGTTGGATA	AATATTAGAG	TTGAACACAC	ACAGATAACA	CACACAGATA	ATATTAATAG	AACGGTTATA	CGTGGGTTGG	TAGTTCAGAT
**iPCR_4 (5′)**	CATCCCTCTA	TAGCTATATG	TACTGTGTGT	GTGGGTAAGG	TTGGACAAGG	ACTCCTGCAT	TATATTGTGT	GCCTCATCGC	TAGTTCAGAT
**iPCR_2 (3′)**	----------	----------	----------	----------	----------	----------	----------	----------	----------
**iPCR_3 (3′)**	----------	----------	----------	----------	----------	----------	----------	----------	----------
**iPCR_4 (3′)**	----------	----------	----------	----------	----------	----------	----------	----------	----------

	280	290	300	310	320	330	340	350	360
**Consensus**	**ATCTACCTTT**	**CCTAGTAGTT**	**ATACCTGTGT**	**G**GACAACACC	TCCTGGGACA	**CACGAATTCT**	**CTCTGAGCAA**	**TCTCTGTTCA**	**ATATCTTTTA**
**CoxI_PCR**	----------	----------	-----TGTGT	GGACAACACC	TCCTGGGACA	CACGAATTCT	CTCTGAGCAA	TCTCTGTTCA	ATATCTTTTA
**Illumina_1**	ATCTACCTTT	CCTAGTAGTT	ATACCTGTGT	GGACAACACC	TCCTGGGACA	CACGAATTCT	CTCTGAGCAA	TCTCTGTTCA	ATATCTTTTA
**Illumina_2**	----------	----------	----------	----------	----------	----------	----------	----------	----------
**Illumina_3**	----------	----------	----------	----------	----------	----------	----------	----------	----------
**Illumina_4**	----------	----------	----------	----------	----------	----------	----------	----------	----------
**Illumina_5**	----------	----------	----------	----------	----------	----------	----------	----------	----------
**iPCR_1 (5′)**	ATCTACCTTT	CCTAGTAGTT	ATACCTGTGT	GGACAACACC	TCCTGGGACA	CACGAATTCT	CTCTGAGCAA	TCTCTGTTCA	ATATCTTTTA
**iPCR_2 (5′)**	ATCTACCTTT	CCTAGTAGTT	ATACCTGTGT	GGACAACACC	TCCTGGGACA	CACGAATTCT	CTCTGAGCAA	TCTCTGTTCA	ATATCTTTTA
**iPCR_3 (5′)**	ATCTACCTTT	CCTAGTAGTT	ATACCTGTGT	GGACAACACC	TCCTGGGACA	CACGAATTCT	CTCTGAGCAA	TCTCTGTTCA	ATATCTTTTA
**iPCR_4 (5′)**	ATCTACCTTT	CCTAGTAGTT	ATACCTGTGT	GGACAACACC	TCCTGGGACA	CACGAATTCT	CTCTGAGCAA	TCTCTGTTCA	ATATCTTTTA
**iPCR_2 (3′)**	----------	----------	----------	----------	----------	----------	----------	----------	----------
**iPCR_3 (3′)**	----------	----------	----------	----------	----------	----------	----------	----------	----------
**iPCR_4 (3′)**	----------	----------	----------	----------	----------	----------	----------	----------	----------

Mitochondrial genome - *Chromera velia* CCAP 1602/1 (KC899110)

```
                ....|....| ....|....| ....|....| ....|....| ....|....| ....|....| ....|....| ....|....| ....|....|
                       370        380        390        400        410        420        430        440        450
Consensus       TTAGTTGTTG TAATATTATA ACCACAAATA CCAAATTTGG CGGCCGTGAT TATTCGGTTG AACTCCCAAA CTGCTTGTAT AAGCGGGCAA
CoxI_PCR        TTAGTTGTTG TAATATTATA ACCACAAATA CCAAATTTGG CGGCCGTGAT TATTCGGTTG AACTCCCAAA CTGCTTGTAT AAGCGGGCAA
Illumina_1      TTAGTTGTTG TAATATTATA ACCACAAATA CCAAATTT-- ---------- ---------- ---------- ---------- ----------
Illumina_2      ---------- -----TTATA ACCACAAATA CCAAATTTGG CGGCCGTGAT TATTCGGTTG AACTCCCAAA CTGCTTGTAT AAGCGGGCAA
Illumina_3      ---------- ---------- ---------- ---------- ---------- ---------- ---------- ---------- ----------
Illumina_4      ---------- ---------- ---------- ---------- ---------- ---------- ---------- ---------- ----------
Illumina_5      ---------- ---------- ---------- ---------- ---------- ---------- ---------- ---------- ----------
iPCR_1 (5')     TTAGTTGTTG TAATATTATA ACCACAAATA CCAAATTTGG CGGCCGTGAT TATTCGGTTG AACTCCCAAA CTGCTTGTAT AAGCGGGCAA
iPCR_2 (5')     TTAGTTGTTG TAATATTATA ACCACAAATA CCAAATTTGG CGGCCGTGAT TATTCGGTTG AACTCCCAAA CTGCTTGTAT AAGCGGGCAA
iPCR_3 (5')     TTAGTTGTTG TAATATTATA ACCACAAATA CCAAATTTGG CGGCCGTGAT TATTCGGTTG AACTCCCAAA CTGCTTGTAT AAGCGGGCAA
iPCR_4 (5')     TTAGTTGTTG TAATATTATA ACCACAAATG CCAAATTTGG CGGCCGTGAT TATTCGGTTG AACTCCCAAA CTGCTTGTAT AAGCGGGCAA
iPCR_2 (3')     ---------- ---------- ---------- ---------- ---------- ---------- ---------- ---------- ----------
iPCR_3 (3')     ---------- ---------- ---------- ---------- ---------- ---------- ---------- ---------- ----------
iPCR_4 (3')     ---------- ---------- ---------- ---------- ---------- ---------- ---------- ---------- ----------
```

```
                ....|....| ....|....| ....|....| ....|....| ....|....| ....|....| ....|....| ....|....| ....|....|
                       460        470        480        490        500        510        520        530        540
Deduced AA                                                    M   F  T   Q  S  E   L  L  A  M   L  M  G   K  P  F
Consensus       TTGGTGTTGT CCAGGGAAAT AATAATCTGA CTCATCATAG TATGTTCACA CAGAGTGAGC TGCTAGCTAT GTTAATGGGA AAACCCTTTG
CoxI_PCR        TTGGTGTTGT CCAGGGAAAT AATAATCTGA CTCATCATAG TATGTTCACA CAGAGTGAGC TGCTAGCTAT GTTAATGGGA AAACCCTTTG
Illumina_1      ---------- ---------- ---------- ---------- ---------- ---------- ---------- ---------- ----------
Illumina_2      TTGGTGTTGT CCAGGGAAAT AATAATCTGA CTCATCATAG TATGTTCACA CAGAGTGAGC TGCTAGCTAT GTTAATGGGA AAACCCTTTG
Illumina_3      ---------- ---------- ---------- ---------- ---------- ---------- ---------- ---------- ----------
Illumina_4      ---------- ---------- ---------- ---------- ---------- ---------- ---------- ---------- ----------
Illumina_5      ---------- ---------- ---------- ---------- ---------- ---------- ---------- ---------- ----------
iPCR_1 (5')     TTGGTGTTGT CCAGGGAGAT AATAATCTGA CTCATCATAG TATGTTCACA CAGAGTGAGC TGCTAGCTAT GTTAATGGGA AAACCCTTTG
iPCR_2 (5')     TTGGTGTTGT CCAGGGAGAT AATAATCTGA CTCATCATAG TATGTTCACA CAGAGTGAGC TGCTAGCTAT GTTAATGGGA AAACCCTTTG
iPCR_3 (5')     TTGGTGTTGT CCAGGGAGAT AATAATCTGA CTCATCATAG TATGTTCACA CAGAGTGAGC TGCTAGCTAT GTTAATGGGA AAACCCTTTG
iPCR_4 (5')     TTGGTGTTGT CCAGGGAAAT AATAATCTGA CTCATCATAG TATGTTCACA CAGAGTGAGC TGCTAGCTAT GTTAATGGGA AAACCCTTTG
iPCR_2 (3')     ---------- ---------- ---------- ---------- ---------- ---------- ---------- ---------- ----------
iPCR_3 (3')     ---------- ---------- ---------- ---------- ---------- ---------- ---------- ---------- ----------
iPCR_4 (3')     ---------- ---------- ---------- ---------- ---------- ---------- ---------- ---------- ----------
```

Mitochondrial genome - *Chromera velia* CCAP 1602/1 (KC899110)

```
                ....|....| ....|....| ....|....| ....|....| ....|....| ....|....| ....|....| ....|....| ....|....|
                          550        560        570        580        590        600        610        620        630
Deduced AA      A  D  N  T   S  M  S   W  C  K   I  N  Q  K   G  S  I   M  F  L   I  N  Y  N   E  Q  T   I  M  N
Consensus       CTGATAACAC TTCGATGAGT TGGTGTAAGA TAAATCAGAA AGGATCAATA ATGTTTCTTA TAAATTATAA TGAACAAACT ATAATGAATA
CoxI_PCR        CTGATAACAC TTCGATGAGT TGGTGTAAGA TAAATCAGAA AGGATCAATA ATGTTTCTTA TAAATTATAA TGAACAAACT ATAATGAATA
Illumina_1      ---------- ---------- ---------- ---------- ---------- ---------- ---------- ---------- ----------
Illumina_2      CTGATAACAC TTCGATGAGT TGGTGTA--- ---------- ---------- ---------- ---------- ---------- ----------
Illumina_3      ---------- ------GAGT TGGTGTAAGA TAAATCAGAA AGGATCAATA ATGTTTCTTA TAAATTATAA TGAACAAACT ATAATGAATA
Illumina_4      ---------- ---------- ---------- ---------- ---------- ---------- ---------- ---------- ----------
Illumina_5      ---------- ---------- ---------- ---------- ---------- ---------- ---------- ---------- ----------
iPCR_1 (5')     CTGATAACAC TTCGATGAGT TGGTGTAAGA TAAATCAGAA AGGATCAATA ATGTTTCTTA TAAATTATAA TGAACAAACT ATAATGAATA
iPCR_2 (5')     CTGATAACAC TTCGATGAGT TGGTGTAAGA TAAATCAGAA AGGATCAATA ATGTTTCTTA TAAATTATAA TGAACAAACT ATAATGAATA
iPCR_3 (5')     CTGATAACAC TTCGATGAGT TGGTGTAAGA TAAATCAGAG AGGATCAATA ATGTTTCTTA TAAATTATAA TGAACAAACT ATAATGAATA
iPCR_4 (5')     CTGATAACAC TTCGATGAGT TGGTGTAAGA TAAATCAGAA AGGATCAATA ATGTTTCTTA TAAATTATAA TGAACAAACT ATAATGAATA
iPCR_2 (3')     ---------- ---------- ---------- ---------- ---------- ---------- ---------- ---------- ----------
iPCR_3 (3')     ---------- ---------- ---------- ---------- ---------- ---------- ---------- ---------- ----------
iPCR_4 (3')     ---------- ---------- ---------- ---------- ---------- ---------- ---------- ---------- ----------
```

```
                ....|....| ....|....| ....|....| ....|....| ....|....| ....|....| ....|....| ....|....| ....|....|
                          640        650        660        670        680        690        700        710        720
Deduced AA      I  D  Y  N   S  N  H   F  M  D   I  F  G  F   S  Y  S   Y  N  E   F  L  Y  L   S  D  S   N  K  I
Consensus       TTGATTATAA TTCCAACCAT TTTATGGATA TATTTGGTTT TTCATATTCT TATAATGAAT TCTTATATTT ATCTGATTCA AATAAAATTA
CoxI_PCR        TTGATTATAA TTCCAACCAT TTTATGGATA TATTTGGTTT TTCATATTCT TATAATGAAT TCTTATATTT ATCTGATTCA AATAAAATTA
Illumina_1      ---------- ---------- ---------- ---------- ---------- ---------- ---------- ---------- ----------
Illumina_2      ---------- ---------- ---------- ---------- ---------- ---------- ---------- ---------- ----------
Illumina_3      TTGATTATAA TTCCAACCAT TTTATGGATA TATTTGGTTT TTCATATTCT TATAATGAAT TCTTATATTT ATCTGATTCA AATAAAATTA
Illumina_4      ---------- ---------- ---------- ---------- ---------- ---------- ---------- ---------- ----------
Illumina_5      ---------- ---------- ---------- ---------- ---------- ---------- ---------- ---------- ----------
iPCR_1 (5')     TTGATTATAA TTCCAACCAT TTTATGGATA TATTTGGTTT TTCATATTCT TATAATGAAT TCTTATATTT ATCTGATTCA AATAAAATTA
iPCR_2 (5')     TTGATTATAA TTCCAACCAT TTTATGGATA TATTTGGTTT TTCATATTCT TATAATGAAT TCTTATATTT ATCTGATTCA AATAAAATTA
iPCR_3 (5')     TTGATTATAA TTCCAACCAT TTTATGGATA TATTTGGTTT TTCATATTCT TATAATGAAT TCTTATATTT ATCTGATTCA AATAAACTTA
iPCR_4 (5')     TTGATTATAA TTCCAACCAT TTTATGGATA TATTTGGTTT TTCATATTCT TATAATGAAT TCTTATATTT ATCTGATTCA AATAAAATTA
iPCR_2 (3')     ---------- ---------- ---------- ---------- ---------- ---------- ---------- ---------- ----------
iPCR_3 (3')     ---------- ---------- ---------- ---------- ---------- ---------- ---------- ---------- ----------
iPCR_4 (3')     ---------- ---------- ---------- ---------- ---------- ---------- ---------- ---------- ----------
```

Mitochondrial genome - *Chromera velia* CCAP 1602/1 (KC899110)

```
               ....|....| ....|....| ....|....| ....|....| ....|....| ....|....| ....|....| ....|....| ....|....|
                     730        740        750        760        770        780        790        800        810
Deduced AA     I  D  N  I   S  T  I   N  H  K   Y  L  G  I   Y  Y  L   Y  Y  G   I  I  F  G   I  I  G   S  I  Y
Consensus      TTGATAACAT TAGTACTATT AATCATAAAT ATTTAGGTAT ATATTATTTA TATTACGGTA TTATCTTTGG TATTATTGGA TCCATATATT
CoxI_PCR       TTGATAACAT TAGTACTATT AATCATAAAT ATTTAGGTAT ATATTATTTA TATTACGGTA TTATCTTTGG TATTATTGGA TCCATATATT
Illumina_1     ---------- ---------- ---------- ---------- ---------- ---------- ---------- ---------- ----------
Illumina_2     ---------- ---------- ---------- ---------- ---------- ---------- ---------- ---------- ----------
Illumina_3     TTGATAACAT TAGTACTATT AATCATAAAT ATTTAGGTAT ATATTATTTA TATTACGGTA TTATCTTTGG TATTATTGGA TCCATATATT
Illumina_4     ---------- ---------- ---------- ---------- ---------- ---------- ---------- ---------- ----------
Illumina_5     ---------- ---------- ---------- ---------- ---------- ---------- ---------- ---------- ----------
iPCR_1 (5')    TTGATAACAT TAGTACTATT AATCATAAAT ATTTAGGTAT ATATTATTTA TATTACGGTA TTATCTTTGG TATTATTGGA TCCATATATT
iPCR_2 (5')    TTGATAACAT TAGTACTATT AATCATAGAT ATTTAGGTAT ATATTATTTA TATTACGGTA TTATCTTTGG TATGATTGGA TCCATATATT
iPCR_3 (5')    TTGATAACAT TAGTACTA-- ---------- ---------- ---------- ---------- ---------- ---------- ----------
iPCR_4 (5')    TTGATAACAT TAGTACTATT AATCATAAAT ATTTGGGTAT ATATTATTTA TATTACGGTA TTATCTTTGG TATTATTGGA TCCATATATT
iPCR_2 (3')    ---------- ---------- ---------- ---------- ---------- ---------- ---------- ---------- ----------
iPCR_3 (3')    ---------- ---------- ---------- ---------- ---------- ---------- ---------- ---------- ----------
iPCR_4 (3')    ---------- ---------- ---------- ---------- ---------- ---------- ---------- ---------- ----------
```

```
               ....|....| ....|....| ....|....| ....|....| ....|....| ....|....| ....|....| ....|....| ....|....|
                     820        830        840        850        860        870        880        890        900
Deduced AA     S  L  I  I   R  I  E   L  G  N   S  N  I  R   I  I  N   Y  N  Y   Y  N  T  V   I  T  I   H  G  L
Consensus      CATTAATTAT TAGAATTGAA CTGGGTAATT CAAATATTAG GATTATCAAT TATAATTATT ATAATACTGT TATAACTATT CATGGATTAT
CoxI_PCR       CATTAATTAT TAGAATTGAA CTGGGTAATT CAAATATTAG GATTATCAAT TATAATTATT ATAATACTGT TATAACTATT CATGGATTAT
Illumina_1     ---------- ---------- ---------- ---------- ---------- ---------- ---------- ---------- ----------
Illumina_2     ---------- ---------- ---------- ---------- ---------- ---------- ---------- ---------- ----------
Illumina_3     CATTAATTAT TAGAATTGAA CTGGGTAATT CAAATATTAG GATTATCAAT TATAATTATT ATAATACTGT TATAACTATT CATGGATTAT
Illumina_4     ---------- ---------- ---------- ---------- ---------- ---------- ----TACTGT TATAACTATT CATGGATTAT
Illumina_5     ---------- ---------- ---------- ---------- ---------- ---------- ---------- ---------- ----------
iPCR_1 (5')    CATTAATTAT TAGAATTGAA CTGGGTAATT CAAATATTAG GATTATCAAT TATAATTATT ATAATACTGT TATAACTATT CATGGATTAT
iPCR_2 (5')    CATTAATTAT TAGAATTGAA CTGGGTAATT CAAATATTAG GATTATCAAT TATAATTATT ATAATACTGT TATAACTATT CATGGATAAT
iPCR_3 (5')    ---------- ---------- ---------- ---------- ---------- ---------- ---------- ---------- ----------
iPCR_4 (5')    CATTAATTAT TAGAATTGAA CTGGGTAATT CAAATATTAG GATTATCAAT TATAATTATT ATAATACTGT TATAACTATT CATGGATTAT
iPCR_2 (3')    ---------- ---------- ---------- ---------- ---------- ---------- ---------- ---------- ----------
iPCR_3 (3')    ---------- ---------- ---------- ---------- ---------- ---------- ---------- ---------- ----------
iPCR_4 (3')    ---------- ---------- ---------- ---------- ---------- ---------- ---------- ---------- ----------
```

Mitochondrial genome - *Chromera velia* CCAP 1602/1 (KC899110)

```
                ....|....| ....|....| ....|....| ....|....| ....|....| ....|....| ....|....| ....|....| ....|....|
                       910        920        930        940        950        960        970        980        990
Deduced AA      L  M  I  F   F  L  I   I  P  V   L  F  S  G   L  G  N   Y  I  S   P  I  N  I   I  K  M   E  I  S
Consensus       TAATGATCTT TTTTCTTATT ATTCCAGTAT TATTTAGTGG ATTAGGAAAT TATATTTCTC CTATTAATAT TATCAAAATG GAAATATCCC
CoxI_PCR        TAATGATCTT TTTTCTTATT ATTCCAGTAT TATTTAGTGG ATTAGGAAAT TATATTTCTC CTATTAATAT TATCAAAATG GAAATATCCC
Illumina_1      ---------- ---------- ---------- ---------- ---------- ---------- ---------- ---------- ----------
Illumina_2      ---------- ---------- ---------- ---------- ---------- ---------- ---------- ---------- ----------
Illumina_3      TAATGATCTT TTTTCTTATT ATTCCAGTAT TATT------ ---------- ---------- ---------- ---------- ----------
Illumina_4      TAATGATCTT TTTTCTTATT ATTCCAGTAT TATTTAGTGG ATTAGGAAAT TATATTTCTC CTATTAATAT TATCAAAATG GAAATATCCC
Illumina_5      ---------- ---------- ---------- ---------- ---------- ---------- ---------- ---------- ----------
iPCR_1 (5')     TAATGATCTT TTTTCTTATT ATTCCAGTAT TATTTA---- ---------- ---------- ---------- ---------- ----------
iPCR_2 (5')     TAACGATCCT TTGTCCTAAT AATCCAGTAT TATTTT---- ---------- ---------- ---------- ---------- ----------
iPCR_3 (5')     ---------- ---------- ---------- ---------- ---------- ---------- ---------- ---------- ----------
iPCR_4 (5')     TAAGGATCTT TTTTCTTATT ATTCCAGTAT TATTTA---- ---------- ---------- ---------- ---------- ----------
iPCR_2 (3')     ---------- ---------- ---------- ---------- ---------- ---------- ---------- ---------- ----------
iPCR_3 (3')     ---------- ---------- ---------- ---------- ---------- ---------- ---------- ---------- ----------
iPCR_4 (3')     ---------- ---------- ---------- ---------- ---------- ---------- ---------- ---------- ----------
```

```
                ....|....| ....|....| ....|....| ....|....| ....|....| ....|....| ....|....| ....|....| ....|....|
                      1000       1010       1020       1030       1040       1050       1060       1070       1080
Deduced AA      L  P  R  V   N  N  L   S  L  I   L  L  L  F   S  F  I   I  F  I   F  N  N  L   I  D  F   G  V  G
Consensus       TTCCGAGAGT GAATAATCTT TCATTAATAT TATTATTATT TTCATTTATT ATTTTTATCT TTAATAATCT TATAGATTTT GGTGTTGGTT
CoxI_PCR        TTCCGAGAGT GAATAATCTT TCATTAATAT TATTATTATT TTCATTTAT- ATTTTTATCT TTAATAATCT TATAGATTTT GGTGTTGGTT
Illumina_1      ---------- ---------- ---------- ---------- ---------- ---------- ---------- ---------- ----------
Illumina_2      ---------- ---------- ---------- ---------- ---------- ---------- ---------- ---------- ----------
Illumina_3      ---------- ---------- ---------- ---------- ---------- ---------- ---------- ---------- ----------
Illumina_4      TTCCGAGAGT GAATAATCTT TCATTAATAT TATTATTATT TTCATTTATT ATTTTTATCT TTAATAATCT TATAGATTTT GGTGTTGGTT
Illumina_5      ---------- ---------- ---------- ---------- ---------- ---------- ---------- ---------- ----------
iPCR_1 (5')     ---------- ---------- ---------- ---------- ---------- ---------- ---------- ---------- ----------
iPCR_2 (5')     ---------- ---------- ---------- ---------- ---------- ---------- ---------- ---------- ----------
iPCR_3 (5')     ---------- ---------- ---------- ---------- ---------- ---------- ---------- ---------- ----------
iPCR_4 (5')     ---------- ---------- ---------- ---------- ---------- ---------- ---------- ---------- ----------
iPCR_2 (3')     ---------- ---------- ---------- ---------- ---------- ---------- ---------- ---------- ----------
iPCR_3 (3')     ---------- ---------- ---------- ---------- ---------- ---------- ---------- ---------- ----------
iPCR_4 (3')     ---------- ---------- ---------- ---------- ---------- ---------- ---------- ---------- ----------
```

Mitochondrial genome - *Chromera velia* CCAP 1602/1 (KC899110)

```
                ....|....| ....|....| ....|....| ....|....| ....|....| ....|....| ....|....| ....|....| ....|....|
                        1090       1100       1110       1120       1130       1140       1150       1160       1170
Deduced AA      L  G  W  T   L  Y  P   P  L  S   T  I  I  Y   L  L  S   I  N  I   I  I  I  G   L  L  V   S  G  I
Consensus       TAGGTTGGAC CCTATACCCT CCATTATCCA CTATTATATA TCTTTTATCG ATCAATATAA TAATTATTGG TTTATTGGTT TCTGGAATTT
CoxI_PCR        TAGGTTGGAC CCTATACCCT CCATTATCCA CTATTATATA TCTTTTATCG ATCAATATAA TAATTATTGG TTTATTGGTT TCTGGAATTT
Illumina_1      ---------- ---------- ---------- ---------- ---------- ---------- ---------- ---------- ----------
Illumina_2      ---------- ---------- ---------- ---------- ---------- ---------- ---------- ---------- ----------
Illumina_3      ---------- ---------- ---------- ---------- ---------- ---------- ---------- ---------- ----------
Illumina_4      TAGGTTGGAC CCTATACCCT CCATTATCCA CTATTATATA TCTTTTATCG ATCAATATAA TAATTATTGG TTTATTGGTT TCTGGAATTT
Illumina_5      ---------- ---------- ---------- ---------- ---------- ---------- ---------- ---------- ----------
iPCR_1 (5')     ---------- ---------- ---------- ---------- ---------- ---------- ---------- ---------- ----------
iPCR_2 (5')     ---------- ---------- ---------- ---------- ---------- ---------- ---------- ---------- ----------
iPCR_3 (5')     ---------- ---------- ---------- ---------- ---------- ---------- ---------- ---------- ----------
iPCR_4 (5')     ---------- ---------- ---------- ---------- ---------- ---------- ---------- ---------- ----------
iPCR_2 (3')     ---------- ---------- ---------- ---------- ---------- ---------- ---------- ---------- ----------
iPCR_3 (3')     ---------- ---------- ---------- ---------- ---------- ---------- ---------- ---------- ----------
iPCR_4 (3')     ---------- ---------- ---------- ---------- ---------- ---------- ---------- ---------- ----------
```

```
                ....|....| ....|....| ....|....| ....|....| ....|....| ....|....| ....|....| ....|....| ....|....|
                        1180       1190       1200       1210       1220       1230       1240       1250       1260
Deduced AA      S  S  I  L   S  S  I   N  F  I   Y  T  I  I   I  I  Y   T  G  L   N  N  S  D   I  N  M   Y  S  T
Consensus       CCAGTATATT ATCTAGTATT AATTTTATTT ATACTATTAT TATTATTTAT ACAGGATTAA ATAATTCCGA TATTAATATG TATTCAACGT
CoxI_PCR        CCAGTATATT ATCTAGTATT AATTTTATTT ATACTATTAT TATTATTTAT ACAGGATTAA ATAATTCCGA TATTAATATG TATTCAACGT
Illumina_1      ---------- ---------- ---------- ---------- ---------- ---------- ---------- ---------- ----------
Illumina_2      ---------- ---------- ---------- ---------- ---------- ---------- ---------- ---------- ----------
Illumina_3      ---------- ---------- ---------- ---------- ---------- ---------- ---------- ---------- ----------
Illumina_4      CCAGTATATT ATCTAGTATT AATTTTATTT ATACTATTAT TATTATTTAT ACAGGATTAA ATAATTCCGA TATTAATATG TATTCAACGT
Illumina_5      ---------- ---------- ---------- ---------- ---------- ---------- ---------- ---------- ----------
iPCR_1 (5')     ---------- ---------- ---------- ---------- ---------- ---------- ---------- ---------- ----------
iPCR_2 (5')     ---------- ---------- ---------- ---------- ---------- ---------- ---------- ---------- ----------
iPCR_3 (5')     ---------- ---------- ---------- ---------- ---------- ---------- ---------- ---------- ----------
iPCR_4 (5')     ---------- ---------- ---------- ---------- ---------- ---------- ---------- ---------- ----------
iPCR_2 (3')     ---------- ---------- ---------- ---------- ---------- ---------- ---------- ---------- ----------
iPCR_3 (3')     ---------- ---------- ---------- ---------- ---------- ---------- ---------- ---------- ----------
iPCR_4 (3')     ---------- ---------- ---------- ---------- ---------- ---------- ---------- ---------- ----------
```

Mitochondrial genome - *Chromera velia* CCAP 1602/1 (KC899110)

```
                ....|....| ....|....| ....|....| ....|....| ....|....| ....|....| ....|....| ....|....| ....|....|
                        1270       1280       1290       1300       1310       1320       1330       1340       1350
Deduced AA      Y  I  G  S   L  L  F   V  S  F   L  L  L  I   V  L  P   L  L  T   T  L  F  I   L  L  I   F  D  L
Consensus       ATATAGGTTC ATTATTATTT GTTTCATTTT TATTATTAAT AGTATTACCA TTATTAACAA CATTATTTAT ATTATTAATA TTTGATTTGA
CoxI_PCR        ATATAGGTTC ATTATTATTT GTTTCATTTT TATTATTAAT AGTATTACCA TTATTAACA- CATTATTTAT ATTATTAATA TTTGATTTGA
Illumina_1      ---------- ---------- ---------- ---------- ---------- ---------- ---------- ---------- ----------
Illumina_2      ---------- ---------- ---------- ---------- ---------- ---------- ---------- ---------- ----------
Illumina_3      ---------- ---------- ---------- ---------- ---------- ---------- ---------- ---------- ----------
Illumina_4      ATATAGGTTC ATTATTATTT GTTTCATTTT TATTATTAAT AGTATTACCA TTATTAACAA CATTATTTAT ATTATTAATA TTTGATTTGA
Illumina_5      ---------- ---------- ---------- ---------- ---------- ---------- ---------- ---------- ----------
iPCR_1 (5’)     ---------- ---------- ---------- ---------- ---------- ---------- ---------- ---------- ----------
iPCR_2 (5’)     ---------- ---------- ---------- ---------- ---------- ---------- ---------- ---------- ----------
iPCR_3 (5’)     ---------- ---------- ---------- ---------- ---------- ---------- ---------- ---------- ----------
iPCR_4 (5’)     ---------- ---------- ---------- ---------- ---------- ---------- ---------- ---------- ----------
iPCR_2 (3’)     ---------- ---------- ---------- ---------- ---------- ---------- ---------- ---------- ----------
iPCR_3 (3’)     ---------- ---------- ---------- ---------- ---------- ---------- ---------- ---------- ----------
iPCR_4 (3’)     ---------- ---------- ---------- ---------- ---------- ---------- ---------- ---------- ----------
```

```
                ....|....| ....|....| ....|....| ....|....| ....|....| ....|....| ....|....| ....|....| ....|....|
                        1360       1370       1380       1390       1400       1410       1420       1430       1440
Deduced AA      N  F  N  T   I  F  F   D  T  Y   S  G  D  V   L  F  F   Q  H  L   F  W  F  F   G  H  P   E  V  Y
Consensus       ATTTTAATAC AATTTTCTTT GATACCTATT CGGGTGATGT TTTATTTTTC CAGCATTTAT TCTGGTTCTT TGGACACCCT GAAGTTTATA
CoxI_PCR        ATTTTAATAC AATTTTCTTT GATACCTATT CGGGTGATGT TTTATTTTTC CAGCATTTAT TCTGGTTCTT TGGACACCCT GAAGTTTATA
Illumina_1      ---------- ---------- ---------- ---------- ---------- ---------- ---------- ---------- ----------
Illumina_2      ---------- ---------- ---------- ---------- ---------- ---------- ---------- ---------- ----------
Illumina_3      ---------- ---------- ---------- ---------- ---------- ---------- ---------- ---------- ----------
Illumina_4      ATTTTAATAC AATTTTCTTT GATACCTATT CGGGTGATGT TTTATTTTTC CAGCATTTAT TCTGGTTCTT TGGACACCCT GAAGTTTATA
Illumina_5      ---------- ---------- ---------- ---------- ---------- ---------- ---------- ---------- ----------
iPCR_1 (5’)     ---------- ---------- ---------- ---------- ---------- ---------- ---------- ---------- ----------
iPCR_2 (5’)     ---------- ---------- ---------- ---------- ---------- ---------- ---------- ---------- ----------
iPCR_3 (5’)     ---------- ---------- ---------- ---------- ---------- ---------- ---------- ---------- ----------
iPCR_4 (5’)     ---------- ---------- ---------- ---------- ---------- ---------- ---------- ---------- ----------
iPCR_2 (3’)     ---------- ---------- ---------- ---------- ---------- ---------- ---------- ---------- ----------
iPCR_3 (3’)     ---------- ---------- ---------- ---------- ---------- ---------- ---------- ---------- ----------
iPCR_4 (3’)     ---------- ---------- ---------- ---------- ---------- ---------- ---------- ---------- ----------
```

Mitochondrial genome - *Chromera velia* CCAP 1602/1 (KC899110)

```
               ....|....| ....|....| ....|....| ....|....| ....|....| ....|....| ....|....| ....|....| ....|....|
                      1450       1460       1470       1480       1490       1500       1510       1520       1530
Deduced AA     I  L  I  L   P  A  F   G  I  I   G  N  T  L   S  V  Y   N  T  R   Y  I  F  G   N  Q  S   M  I  T
Consensus      TTCTTATATT ACCAGCATTT GGAATTATAG GTAATACTTT ATCTGTTTAT AATACCCGAT ATATTTTTGG AAATCAATCT ATGATTACTG
CoxI_PCR       TTCTTATATT ACCAGCATTT GGAATTATAG GTAATACTTT ATCTGTTTAT AATACCCGAT ATATTTTTGG AAATCAATCT ATGATTACTG
Illumina_1     ---------- ---------- ---------- ---------- ---------- ---------- ---------- ---------- ----------
Illumina_2     ---------- ---------- ---------- ---------- ---------- ---------- ---------- ---------- ----------
Illumina_3     ---------- ---------- ---------- ---------- ---------- ---------- ---------- ---------- ----------
Illumina_4     TTCTTATATT ACCAGCATTT GGAATTATAG GTAATACTTT ATCTGTTTAT AATACCCGAT ATATTTTTGG AAATCAATCT ATGATTACTG
Illumina_5     ---------- ---------- ---------- ---------- ---------- ---------- ---------- ---------- ----------
iPCR_1 (5’)    ---------- ---------- ---------- ---------- ---------- ---------- ---------- ---------- ----------
iPCR_2 (5’)    ---------- ---------- ---------- ---------- ---------- ---------- ---------- ---------- ----------
iPCR_3 (5’)    ---------- ---------- ---------- ---------- ---------- ---------- ---------- ---------- ----------
iPCR_4 (5’)    ---------- ---------- ---------- ---------- ---------- ---------- ---------- ---------- ----------
iPCR_2 (3’)    ---------- ---------- ---------- ---------- ---------- ---------- ---------- ---------- ----------
iPCR_3 (3’)    ---------- ---------- ---------- ---------- ---------- ---------- ---------- ---------- ----------
iPCR_4 (3’)    ---------- ---------- ---------- ---------- ---------- ---------- ---------- ---------- ----------
```

```
               ....|....| ....|....| ....|....| ....|....| ....|....| ....|....| ....|....| ....|....| ....|....|
                      1540       1550       1560       1570       1580       1590       1600       1610       1620
Deduced AA     A  M  G  C   I  S  T   I  G  L   A  V  W  G   H  H  M   Y  S  V   G  L  L  T   D  T  R   S  Y  F
Consensus      CAATGGGTTG TATTTCCACT ATAGGACTCG CGGTTTGGGG ACATCACATG TACAGTGTTG GGTTGTTAAC TGATACCAGA TCCTATTTCA
CoxI_PCR       CAATGGGTTG TATTTCCACT ATAGGACTCG CGGTTTGGGG ACATCACATG TACAGTGTTG GGTTGTTAAC TGATACCAGA TCCTATTTCA
Illumina_1     ---------- ---------- ---------- ---------- ---------- ---------- ---------- ---------- ----------
Illumina_2     ---------- ---------- ---------- ---------- ---------- ---------- ---------- ---------- ----------
Illumina_3     ---------- ---------- ---------- ---------- ---------- ---------- ---------- ---------- ----------
Illumina_4     CAATGGGTTG TATTTCCACT ATAGGACTCG CGGTTTGGGG ACATCACATG TACAGTGTTG GGTTGTTAAC TGATACCAGA TCCTATTTCA
Illumina_5     ---------- ---------- ---------- ---------- ---------- ---------- ---------- ---------- ----------
iPCR_1 (5’)    ---------- ---------- ---------- ---------- ---------- ---------- ---------- ---------- ----------
iPCR_2 (5’)    ---------- ---------- ---------- ---------- ---------- ---------- ---------- ---------- ----------
iPCR_3 (5’)    ---------- ---------- ---------- ---------- ---------- ---------- ---------- ---------- ----------
iPCR_4 (5’)    ---------- ---------- ---------- ---------- ---------- ---------- ---------- ---------- ----------
iPCR_2 (3’)    ---------- ---------- ---------- ---------- ---------- ---------- ---------- ---------- ----------
iPCR_3 (3’)    ---------- ---------- ---------- ---------- ---------- ---------- ---------- ---------- ----------
iPCR_4 (3’)    ---------- ---------- ---------- ---------- ---------- ---------- ---------- ---------- ----------
```

Mitochondrial genome - *Chromera velia* CCAP 1602/1 (KC899110)

```
                 ....|....| ....|....| ....|....| ....|....| ....|....| ....|....| ....|....| ....|....| ....|....|
                         1630       1640       1650       1660       1670       1680       1690       1700       1710
Deduced AA       T  F  I  T   I  L  I   S  I  P   T  G  T  K   L  F  N   W  Y  S   T  L  L  H   S  F  I   I  N  N
Consensus        CTTTTATTAC TATTCTTATT AGTATACCAA CCGGAACCAA ATTATTCAAT TGGTACTCCA CATTATTACA TTCATTTATT ATTAATAATT
CoxI_PCR         CTTTTATTAC TATTCTTATT AGTATACCAA CCGGAACCAA ATTATTCAAT TGGTACTCCA CATTATTACA TTCATTTATT ATTAATAATT
Illumina_1       ---------- ---------- ---------- ---------- ---------- ---------- ---------- ---------- ----------
Illumina_2       ---------- ---------- ---------- ---------- ---------- ---------- ---------- ---------- ----------
Illumina_3       ---------- ---------- ---------- ---------- ---------- ---------- ---------- ---------- ----------
Illumina_4       CTTTTATTAC TATTCTTATT AGTATACCAA CCGGAACCAA ATTATTCAAT TGGTACTCCA CATTATTACA TTCATTTATT ATTAATAATT
Illumina_5       ---------- ---------- ---------- ---------- ---------- ---------- ---------- ---------- ----------
iPCR_1 (5’)      ---------- ---------- ---------- ---------- ---------- ---------- ---------- ---------- ----------
iPCR_2 (5’)      ---------- ---------- ---------- ---------- ---------- ---------- ---------- ---------- ----------
iPCR_3 (5’)      ---------- ---------- ---------- ---------- ---------- ---------- ---------- ---------- ----------
iPCR_4 (5’)      ---------- ---------- ---------- ---------- ---------- ---------- ---------- ---------- ----------
iPCR_2 (3’)      ---------- ---------- ---------- ---------- ---------- ---------- ---------- ---------- ----------
iPCR_3 (3’)      ---------- ---------- ---------- ---------- ---------- ---------- ---------- ---------- ----------
iPCR_4 (3’)      ---------- ---------- ---------- ---------- ---------- ---------- ---------- ---------- ----------
```

```
                 ....|....| ....|....| ....|....| ....|....| ....|....| ....|....| ....|....| ....|....| ....|....|
                         1720       1730       1740       1750       1760       1770       1780       1790       1800
Deduced AA       Y  L  N  I   Y  C  L   F  F  L   L  S  F  L   L  G  G   V  T  G   V  I  L  G   N  S  L   V  D  L
Consensus        ATCTTAATAT TTATTGTTTA TTCTTTTTAT TATCATTCTT ATTAGGTGGA GTTACCGGGG TTATACTTGG GAATTCATTA GTAGATTTGG
CoxI_PCR         ATCTTAATAT TTATTGTTTA TTCTTTTTAT TATCATTCTT ATTAGGTGGA GTTACCGGGG TTATACTTGG GAATTCATTA GTAGATTTGG
Illumina_1       ---------- ---------- ---------- ---------- ---------- ---------- ---------- ---------- ----------
Illumina_2       ---------- ---------- ---------- ---------- ---------- ---------- ---------- ---------- ----------
Illumina_3       ---------- ---------- ---------- ---------- ---------- ---------- ---------- ---------- ----------
Illumina_4       ATCTTAATAT TTATTGTTTA TTCTTTTTAT TATCATTCTT ATTAGGTGGA GTTACCGGGG TTATACTTGG GAATTCATTA GTAGATTTGG
Illumina_5       ---------- ---------- ---------- ---------- ---------- ---------- ---------- ---------- ----------
iPCR_1 (5’)      ---------- ---------- ---------- ---------- ---------- ---------- ---------- ---------- ----------
iPCR_2 (5’)      ---------- ---------- ---------- ---------- ---------- ---------- ---------- ---------- ----------
iPCR_3 (5’)      ---------- ---------- ---------- ---------- ---------- ---------- ---------- ---------- ----------
iPCR_4 (5’)      ---------- ---------- ---------- ---------- ---------- ---------- ---------- ---------- ----------
iPCR_2 (3’)      ---------- ---------- ---------- ---------- ---------- ---------- ---------- ---------- ----------
iPCR_3 (3’)      ---------- ---------- ---------- ---------- ---------- ---------- ---------- ---------- ----------
iPCR_4 (3’)      ---------- ---------- ---------- ---------- ---------- ---------- ---------- ---------- ----------
```

Mitochondrial genome - *Chromera velia* CCAP 1602/1 (KC899110)

```
               ....|....| ....|....| ....|....| ....|....| ....|....| ....|....| ....|....| ....|....| ....|....|
                        1810       1820       1830       1840       1850       1860       1870       1880       1890
Deduced AA     A  L  H  D   T  Y  Y   V  V  G   H  F  H  V   V  L  S   V  S  A   I  I  T  I   Y  T  A   I  L  S
Consensus      CACTTCATGA TACTTATTAT GTTGTCGGTC ATTTCCACGT GGTTCTCTCA GTTTCTGCAA TTATAACCAT TTATACAGCT ATTTTATCTT
CoxI_PCR       CACTTCATGA TACTTATTAT GTTGTCGGTC ATTTCCACGT GGTTCTCTCA GTTTCTGCAA TTATAACCAT TTATACAGCT ATTTTATCTT
Illumina_1     ---------- ---------- ---------- ---------- ---------- ---------- ---------- ---------- ----------
Illumina_2     ---------- ---------- ---------- ---------- ---------- ---------- ---------- ---------- ----------
Illumina_3     ---------- ---------- ---------- ---------- ---------- ---------- ---------- ---------- ----------
Illumina_4     CACTTCATGA TACTTATTAT GTTGTCGGTC ATTTCCACGT GGTTCTCTCA GTTTCTGCAA TTATAACCAT TTATACAGCT ATTTTATCTT
Illumina_5     ---------- ---------- ---------- ---------- ---------- ---------- ---------- ---------- ----------
iPCR_1 (5')    ---------- ---------- ---------- ---------- ---------- ---------- ---------- ---------- ----------
iPCR_2 (5')    ---------- ---------- ---------- ---------- ---------- ---------- ---------- ---------- ----------
iPCR_3 (5')    ---------- ---------- ---------- ---------- ---------- ---------- ---------- ---------- ----------
iPCR_4 (5')    ---------- ---------- ---------- ---------- ---------- ---------- ---------- ---------- ----------
iPCR_2 (3')    ---------- ---------- ---------- ---------- ---------- ---------- ---------- ---------- ----------
iPCR_3 (3')    ---------- ---------- ---------- ---------- ---------- ---------- ---------- ---------- ----------
iPCR_4 (3')    ---------- ---------- ---------- ---------- ---------- ---------- ---------- ---------- ----------
```

```
               ....|....| ....|....| ....|....| ....|....| ....|....| ....|....| ....|....| ....|....| ....|....|
                        1900       1910       1920       1930       1940       1950       1960       1970       1980
Deduced AA     Y  L  E  V   L  F  Y   N  S  T   S  S  V  V   N  N  S   F  N  I   I  Y  H  Y   L  I  Y   G  L  G
Consensus      ATTTAGAGGT TTTATTTTAT AACTCGACAA GTTCGGTTGT TAATAACTCT TTCAATATTA TATATCATTA TCTTATATAT GGATTAGGTT
CoxI_PCR       ATTTAGAGGT TTTATTTTAT AACTCGACAA GTTCGGTTGT TAATAACTCT TTCAATATTA TATATCATTA TCTTATATAT GGATTAGGTT
Illumina_1     ---------- ---------- ---------- ---------- ---------- ---------- ---------- ---------- ----------
Illumina_2     ---------- ---------- ---------- ---------- ---------- ---------- ---------- ---------- ----------
Illumina_3     ---------- ---------- ---------- ---------- ---------- ---------- ---------- ---------- ----------
Illumina_4     ATTTAGAGGT TTTATTTTAT AACTCGACAA GTTCGGTTGT TAATAACTCT TTCAATATTA TATATCATTA TCTTATATAT GGATTAGGTT
Illumina_5     ---------- ---------- ---------- ---------- ---------- ---------- ---------- ---------- ----------
iPCR_1 (5')    ---------- ---------- ---------- ---------- ---------- ---------- ---------- ---------- ----------
iPCR_2 (5')    ---------- ---------- ---------- ---------- ---------- ---------- ---------- ---------- ----------
iPCR_3 (5')    ---------- ---------- ---------- ---------- ---------- ---------- ---------- ---------- ----------
iPCR_4 (5')    ---------- ---------- ---------- ---------- ---------- ---------- ---------- ---------- ----------
iPCR_2 (3')    ---------- ---------- ---------- ---------- ---------- ---------- ---------- ---------- ----------
iPCR_3 (3')    ---------- ---------- ---------- ---------- ---------- ---------- ---------- ---------- --ATTAGGTT
iPCR_4 (3')    ---------- ---------- ---------- ---------- ---------- ---------- ---------- ---------- ----TAGGTT
```

Mitochondrial genome - *Chromera velia* CCAP 1602/1 (KC899110)

```
                ....|....| ....|....| ....|....| ....|....| ....|....| ....|....| ....|....| ....|....| ....|....|
                          1990       2000       2010       2020       2030       2040       2050       2060       2070
Deduced AA      L  C  G  L   F  I  P   M  H  Y   S  G  L  F   T  L  P   R  R  I   P  D  L  I   D  G  L   N  C  W
Consensus       TATGCGGTCT GTTTATTCCT ATGCATTACT CAGGATTATT CACTTTACCC CGAAGAATTC CTGATTTAAT CGATGGTTTG AATTGTTGGA
CoxI_PCR        TATGCGGTCT GTTTATTCCT ATGCATTACT CAGGATTATT CACTTTACCC CGAAGAATTC CTGATTTAAT CGATGGTTTG AATTGTTGGA
Illumina_1      ---------- ---------- ---------- ---------- ---------- ---------- ---------- ---------- ----------
Illumina_2      ---------- ---------- ---------- ---------- ---------- ---------- ---------- ---------- ----------
Illumina_3      ---------- ---------- ---------- ---------- ---------- ---------- ---------- ---------- ----------
Illumina_4      TATGCGGTCT GTTTATTCCT ATGCATTACT CAGGATTATT CACTTTACCC CGAAGAATTC CTGATTTA-- ---------- ----------
Illumina_5      ---------- ---------- ---------- ---------- ---------- ---------- ---------- ---------- --TTGTTGGA
iPCR_1 (5')     ---------- ---------- ---------- ---------- ---------- ---------- ---------- ---------- ----------
iPCR_2 (5')     ---------- ---------- ---------- ---------- ---------- ---------- ---------- ---------- ----------
iPCR_3 (5')     ---------- ---------- ---------- ---------- ---------- ---------- ---------- ---------- ----------
iPCR_4 (5')     ---------- ---------- ---------- ---------- ---------- ---------- ---------- ---------- ----------
iPCR_2 (3')     ---------- ---------- ---------- ---------- ---------- -------TTC CTGATTTAAT CGATGGTTTG AATTGTTGGA
iPCR_3 (3')     TATGCGGTCT GTTTATTCCT ATGCATTACT CAGGATTATT CACTTTACCC CGAAGAATTC CTGATTTAAT CGATGGTTTG AATTGTTGGA
iPCR_4 (3')     TATGCGGTCT GTTTATTCCT ATGCATTACT CAGGATTATT CACTTTACCC CGAGGAATTC CTGATTTAAT CGATGGTTTG AATTGTTGGA
```

```
                ....|....| ....|....| ....|....| ....|....| ....|....| ....|....| ....|....| ....|....| ....|....|
                          2080       2090       2100       2110       2120       2130       2140       2150       2160
Deduced AA      N  S  L  S   S  L  T   S  G  I   V  F  C  S   F  S  V   L  T  P   Y  N  P  I   I  N  I   D  E  *
Consensus       ACAGTTTATC GTCTCTAACA TCAGGTATAG TTTTCTGTTC ATTCTCTGTG TTGACCCCGT ATAATCCAAT AATTAACATC GATGAATAGT
CoxI_PCR        ACAGTTTATC GTCTCTAACA TCAGGTATAG TTTTCTGTTC ATTCTCTGTG TTGACCCCGT ATAATCCAAT AATTAACATC GATGAATAGT
Illumina_1      ---------- ---------- ---------- ---------- ---------- ---------- ---------- ---------- ----------
Illumina_2      ---------- ---------- ---------- ---------- ---------- ---------- ---------- ---------- ----------
Illumina_3      ---------- ---------- ---------- ---------- ---------- ---------- ---------- ---------- ----------
Illumina_4      ---------- ---------- ---------- ---------- ---------- ---------- ---------- ---------- ----------
Illumina_5      ACAGTTTATC GTCTCTAACA TCAGGTATAG TTTTCTGTTC ATTCTCTGTG TTGACCCCGT ATAATCCAAT AATTAACATC GATGAATAGT
iPCR_1 (5')     ---------- ---------- ---------- ---------- ---------- ---------- ---------- ---------- ----------
iPCR_2 (5')     ---------- ---------- ---------- ---------- ---------- ---------- ---------- ---------- ----------
iPCR_3 (5')     ---------- ---------- ---------- ---------- ---------- ---------- ---------- ---------- ----------
iPCR_4 (5')     ---------- ---------- ---------- ---------- ---------- ---------- ---------- ---------- ----------
iPCR_2 (3')     ACAGTTTATC GTCTCTAACA TCAGGTATAG TTTTCTGTTC ATTCTCTGTG TTGACCCCGT ATAATCCAAT AATTAACATC GATGAATAGT
iPCR_3 (3')     ACAGTTTATC GTCTCTAACA TCAGGTATAG TTTTCTGTTC ATTCTCTGTG TTGACCCCGT ATAATCCAAT AATTAACATC GATGAATAGT
iPCR_4 (3')     ACAGTTTATC GTCTCTAACA TCAGGTATAG TTTTCTGTTC ATTCTCTGTG TTGACCCCGT ATAATCCAAT AATTAACATC GATGAATAGT
```

Mitochondrial genome - *Chromera velia* CCAP 1602/1 (KC899110)

```
                ....|....| ....|....| ....|....| ....|....| ....|....| ....|....| ....|....| ....|....| ....|....|
                        2170       2180       2190       2200       2210       2220       2230       2240       2250
Consensus       TGCTGGGTTA GCTAGTGATT TGGTATGTAA GAGGAATGCC TGTATGGGAC AGTCGTATGC CGGGTTATAA C--------- ----------
CoxI_PCR        ATCTGGGTTA GCTAGTGATT TGGTATGTAA GAGGAATGCC TGTATGGGAC AGTCGTATGC CGGGT----- ---------- ----------
Illumina_1      ---------- ---------- ---------- ---------- ---------- ---------- ---------- ---------- ----------
Illumina_2      ---------- ---------- ---------- ---------- ---------- ---------- ---------- ---------- ----------
Illumina_3      ---------- ---------- ---------- ---------- ---------- ---------- ---------- ---------- ----------
Illumina_4      ---------- ---------- ---------- ---------- ---------- ---------- ---------- ---------- ----------
Illumina_5      TGCTGGGTTA GCTAGTGATT TGGTATGTAA GAGGAATGCC TGTATGGGAC AGTCGTATGC CGGGTTATAA C--------- ----------
iPCR_1 (5')     ---------- ---------- ---------- ---------- ---------- ---------- ---------- ---------- ----------
iPCR_2 (5')     ---------- ---------- ---------- ---------- ---------- ---------- ---------- ---------- ----------
iPCR_3 (5')     ---------- ---------- ---------- ---------- ---------- ---------- ---------- ---------- ----------
iPCR_4 (5')     ---------- ---------- ---------- ---------- ---------- ---------- ---------- ---------- ----------
iPCR_2 (3')     TGCTGGGTTA GCTAGTGATT TGGTATGTAA GAGGAATGCC TGTATGGGAC AGTCGTATGC CGGGTTATAA CAGACCAACA ACACCTTCTG
iPCR_3 (3')     TGCTGGGTTA GCTAGTGATT TGGTATGTAA GAGGAATGCC TGTATGGGAC AGTCGTATGC CGGGTTATAA CAGACCAACA ACACCTTCTG
iPCR_4 (3')     TGCTGGGTTA GCTAGTGATT TGGTATGTAA GAGGAATGCC TGTATGGGAC AGTCGTATGC CGGGTTATAA CAGACCAACA GCACCTTCTG
```

```
                ....|....| ....|....| ....|....| ....|....| ....|....| ....|....| ....|....| ....|....| ....|....|
                        2260       2270       2280       2290       2300       2310       2320       2330       2340
Consensus       ---------- ---------- ---------- ---------- ---------- ---------- ---------- ---------- ----------
CoxI_PCR        ---------- ---------- ---------- ---------- ---------- ---------- ---------- ---------- ----------
Illumina_1      ---------- ---------- ---------- ---------- ---------- ---------- ---------- ---------- ----------
Illumina_2      ---------- ---------- ---------- ---------- ---------- ---------- ---------- ---------- ----------
Illumina_3      ---------- ---------- ---------- ---------- ---------- ---------- ---------- ---------- ----------
Illumina_4      ---------- ---------- ---------- ---------- ---------- ---------- ---------- ---------- ----------
Illumina_5      ---------- ---------- ---------- ---------- ---------- ---------- ---------- ---------- ----------
iPCR_1 (5')     ---------- ---------- ---------- ---------- ---------- ---------- ---------- ---------- ----------
iPCR_2 (5')     ---------- ---------- ---------- ---------- ---------- ---------- ---------- ---------- ----------
iPCR_3 (5')     ---------- ---------- ---------- ---------- ---------- ---------- ---------- ---------- ----------
iPCR_4 (5')     ---------- ---------- ---------- ---------- ---------- ---------- ---------- ---------- ----------
iPCR_2 (3')     TTCATCCCTC TATAGCTATA TGTACTGTGT GTGTGGGATT ATTGGTAGAA CACGGTTATT CATTATCTAT GCCCACATGC CTGTGGGAGT
iPCR_3 (3')     TTCATCCCTC TATAGCTATA TGTACTGTGT GTGTGGGATT ATTGGTAGAA CACGGTTATT CATTATCTAT GCCCACATGC CTGTGGGAGT
iPCR_4 (3')     TTCATCCCTC TATAGCTATA TGTACTGTGT GTGTGGGTAA GGTTGGACAA GGACTCCTGC ATTATATTGT GTGCCTCATC GCTAGTTCAG
```

Mitochondrial genome - *Chromera velia* CCAP 1602/1 (KC899110)

```
                ....|....| ....|....| ....|....| ....|....| ....|....| ....|....| ....|....| ....|....| ....|....|
                      2350       2360       2370       2380       2390       2400       2410       2420       2430
Consensus       ---------- ---------- ---------- ---------- ---------- ---------- ---------- ---------- ----------
CoxI_PCR        ---------- ---------- ---------- ---------- ---------- ---------- ---------- ---------- ----------
Illumina_1      ---------- ---------- ---------- ---------- ---------- ---------- ---------- ---------- ----------
Illumina_2      ---------- ---------- ---------- ---------- ---------- ---------- ---------- ---------- ----------
Illumina_3      ---------- ---------- ---------- ---------- ---------- ---------- ---------- ---------- ----------
Illumina_4      ---------- ---------- ---------- ---------- ---------- ---------- ---------- ---------- ----------
Illumina_5      ---------- ---------- ---------- ---------- ---------- ---------- ---------- ---------- ----------
iPCR_1 (5')     ---------- ---------- ---------- ---------- ---------- ---------- ---------- ---------- ----------
iPCR_2 (5')     ---------- ---------- ---------- ---------- ---------- ---------- ---------- ---------- ----------
iPCR_3 (5')     ---------- ---------- ---------- ---------- ---------- ---------- ---------- ---------- ----------
iPCR_4 (5')     ---------- ---------- ---------- ---------- ---------- ---------- ---------- ---------- ----------
iPCR_2 (3')     GTTGGATAAA TATTAGAGTT GAACACACAC AGATAACACA CACAGATAAT ATTAATAGAA CGGTTATACG TGGGTTGGTA GTTCAGATAT
iPCR_3 (3')     GTTGGATAAA TATTAGAGTT GAACACACAC AGATAACACA CACAGATAAT ATTAATAGAA CGGTTATACG TGGGTTGGTA GTTCAGATAT
iPCR_4 (3')     ATATCTACCT TTCCTAGTAG TTATACCTGT GTGGACAACA CCTCCTGGGA CACACGAATT CTCTCTGAGC AATCTCTGTT CAATATCTTT
```

```
                ....|....| ....|....| ....|....| ....|....| ....|....| ....|....| ....|....| ....|....| ....|....|
                      2440       2450       2460       2470       2480       2490       2500       2510       2520
Consensus       ---------- ---------- ---------- ---------- ---------- ---------- ---------- ---------- ----------
CoxI_PCR        ---------- ---------- ---------- ---------- ---------- ---------- ---------- ---------- ----------
Illumina_1      ---------- ---------- ---------- ---------- ---------- ---------- ---------- ---------- ----------
Illumina_2      ---------- ---------- ---------- ---------- ---------- ---------- ---------- ---------- ----------
Illumina_3      ---------- ---------- ---------- ---------- ---------- ---------- ---------- ---------- ----------
Illumina_4      ---------- ---------- ---------- ---------- ---------- ---------- ---------- ---------- ----------
Illumina_5      ---------- ---------- ---------- ---------- ---------- ---------- ---------- ---------- ----------
iPCR_1 (5')     ---------- ---------- ---------- ---------- ---------- ---------- ---------- ---------- ----------
iPCR_2 (5')     ---------- ---------- ---------- ---------- ---------- ---------- ---------- ---------- ----------
iPCR_3 (5')     ---------- ---------- ---------- ---------- ---------- ---------- ---------- ---------- ----------
iPCR_4 (5')     ---------- ---------- ---------- ---------- ---------- ---------- ---------- ---------- ----------
iPCR_2 (3')     CTACCTTTCC TAGTAGTTAT ACCTGTGTGG ACAACACCTC CTGGGACACA CGAATTCTCT CTGAGCAATC TCTGTTCAAT ATCTTTTATT
iPCR_3 (3')     CTACCTTTCC TAGTAGTTAT ACCTGTGTGG ACAACACCTC CTGGGACACA CGAATTCTCT CTGAGCAATC TCTGTTCAAT ATCTTTTATT
iPCR_4 (3')     TATTAGTTGT TGTAATATTA TAACCACAAA TGCCAAATTT GGCGGCCGTG ATTATTCGGT TGAACTCCCA AACTGCTTGT ATAAGCGGGC
```

Mitochondrial genome - *Chromera velia* CCAP 1602/1 (KC899110)

```
                ....|....| ....|....| ....|....| ....|....| ....|....| ....|....| ....|....| ....|....| ....|....|
                        2530       2540       2550       2560       2570       2580       2590       2600       2610
Consensus       ---------- ---------- ---------- ---------- ---------- ---------- ---------- ---------- ----------
CoxI_PCR        ---------- ---------- ---------- ---------- ---------- ---------- ---------- ---------- ----------
Illumina_1      ---------- ---------- ---------- ---------- ---------- ---------- ---------- ---------- ----------
Illumina_2      ---------- ---------- ---------- ---------- ---------- ---------- ---------- ---------- ----------
Illumina_3      ---------- ---------- ---------- ---------- ---------- ---------- ---------- ---------- ----------
Illumina_4      ---------- ---------- ---------- ---------- ---------- ---------- ---------- ---------- ----------
Illumina_5      ---------- ---------- ---------- ---------- ---------- ---------- ---------- ---------- ----------
iPCR_1 (5')     ---------- ---------- ---------- ---------- ---------- ---------- ---------- ---------- ----------
iPCR_2 (5')     ---------- ---------- ---------- ---------- ---------- ---------- ---------- ---------- ----------
iPCR_3 (5')     ---------- ---------- ---------- ---------- ---------- ---------- ---------- ---------- ----------
iPCR_4 (5')     ---------- ---------- ---------- ---------- ---------- ---------- ---------- ---------- ----------
iPCR_2 (3')     AGTTGTTGTA ATATTATAAC CACAAATACC AAATTTGGCG GCCGTGATTA TTCGGTTGAA CTCCCAAACT GCTTGTATAA GCGGGCAATT
iPCR_3 (3')     AGTTGTTGTA ATATTATAAC CACAAATACC AAATTTGGCG GCCGTGATTA TTCGGTTGAA CTCCCAAACT GCTTGTATAA GCGGGCAATT
iPCR_4 (3')     AATTGGTGTT GTCCAGGGAA ATAATAATCT GACTCATCAT AGT------- ---------- ---------- ---------- ----------
```

```
                ....|....| ....|....| ....|....| ....|....
                        2620       2630       2640
Consensus       ---------- ---------- ---------- ---------
CoxI_PCR        ---------- ---------- ---------- ---------
Illumina_1      ---------- ---------- ---------- ---------
Illumina_2      ---------- ---------- ---------- ---------
Illumina_3      ---------- ---------- ---------- ---------
Illumina_4      ---------- ---------- ---------- ---------
Illumina_5      ---------- ---------- ---------- ---------
iPCR_1 (5')     ---------- ---------- ---------- ---------
iPCR_2 (5')     ---------- ---------- ---------- ---------
iPCR_3 (5')     ---------- ---------- ---------- ---------
iPCR_4 (5')     ---------- ---------- ---------- ---------
iPCR_2 (3')     GGTGTTGTCC AGGGAGATAA TAATCTGACT CATCATAGT
iPCR_3 (3')     GGTGTTGTCC AGGGAGATAA TAATCTGACT CATCATAGT
iPCR_4 (3')     ---------- ---------- ---------- ---------
```

Figure S1: Alignment of the mitochondrial genome from *Chromera velia* CCAP 1602/1 based on five genomic nodes of the metagenome sequencing project (BioProject: PRJNA196886; Illumina_1 to Illumina_5 [Node186709, Node235449, Node461926, Node2236, Node 119218]) and four sequences obtained by inverse PCR amplification (iPCR_1 to iPCR_4). The deduced amino acid sequence of the sole mitochondrial gene, *cox*I, is highlighted in yellow, the start and stop codon in green and red, respectively. Overlapping Illumina sequences are shown in light gray. Homologous regions in variable alignment positions between Illumina and iPCR as well as iPCR regions are shown in light green and light red, respectively. The complete *cox*I gene was amplified with the two primers highlighted in light blue; the PCR-product was cloned and resequenced. The nucleotide consensus sequence of all genome and PCR sequences, which has been deposited under the accession number KC899110, is shown in bold.
