# Supplementary figures and images for: Chromera velia, Endosymbioses and the Rhodoplex Hypothesis—Plastid Evolution in Cryptophytes, Alveolates, Stramenopiles, and Haptophytes (CASH Lineages)
Source: Genome Biol Evol. 2014 Feb 25;6(3):666–84. doi: 10.1093/gbe/evu043 (PMC3971594; doi:10.1093/gbe/evu043)

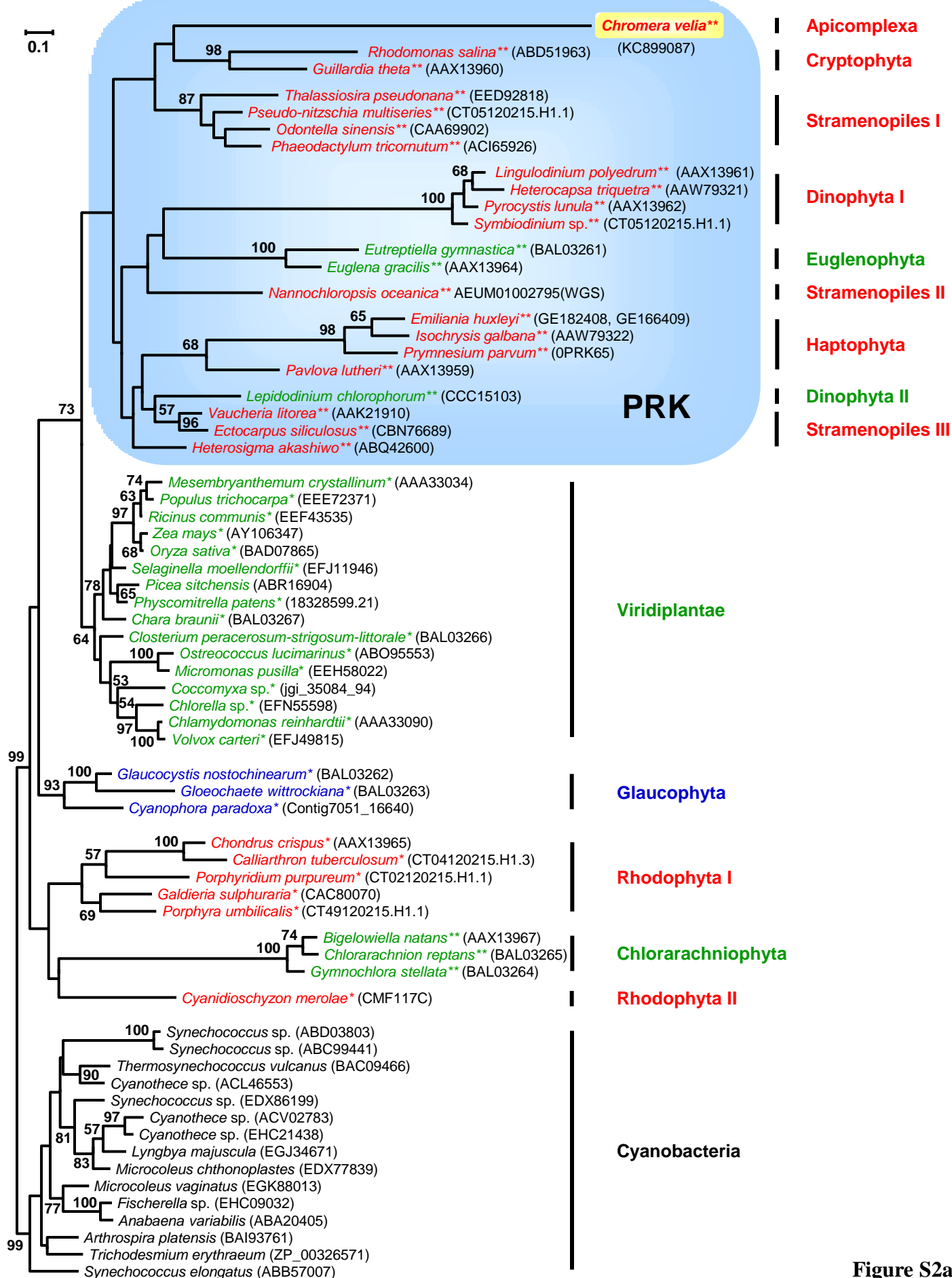

Figure S2a

PRK

0.1

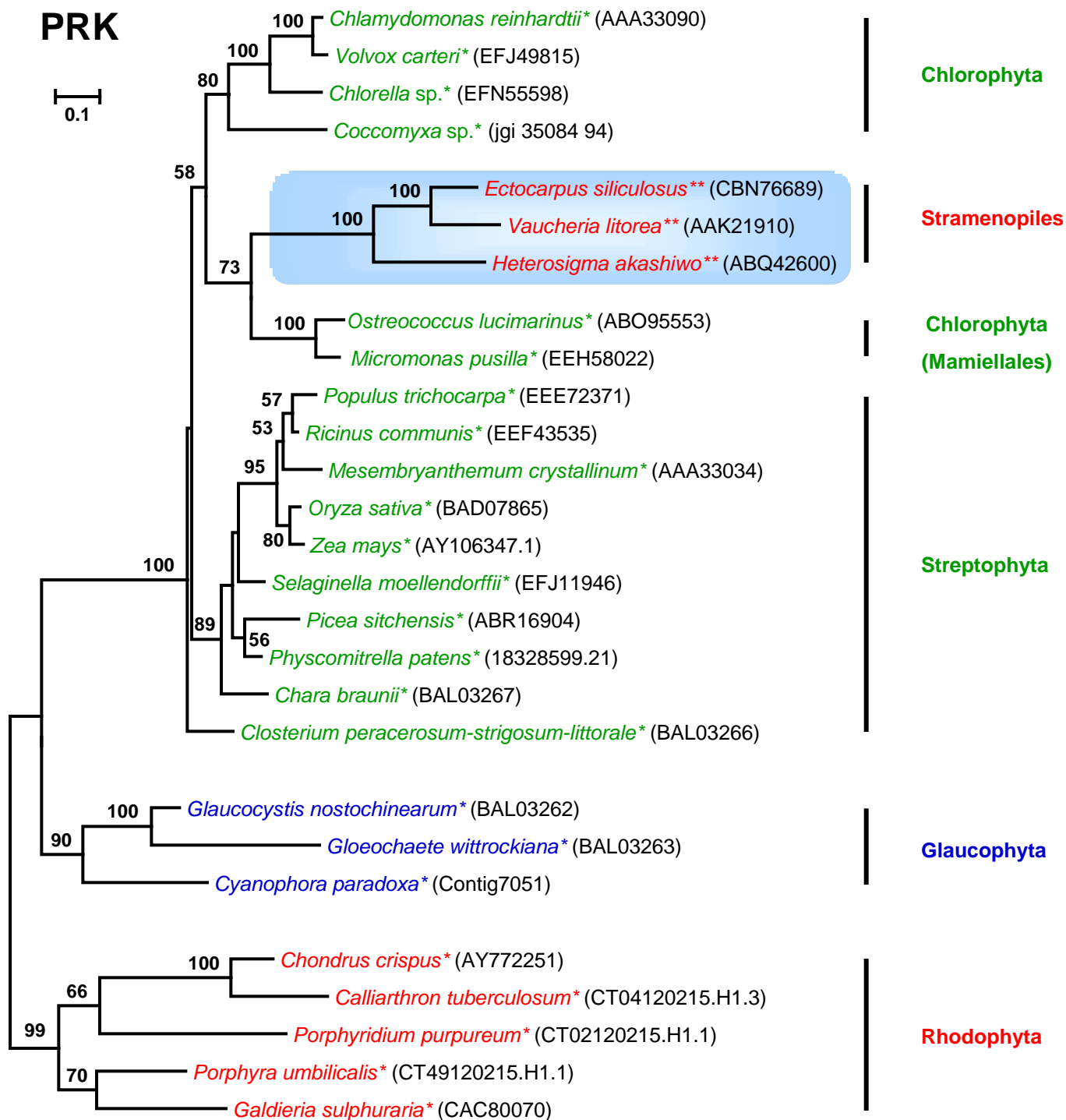

Figure S2b

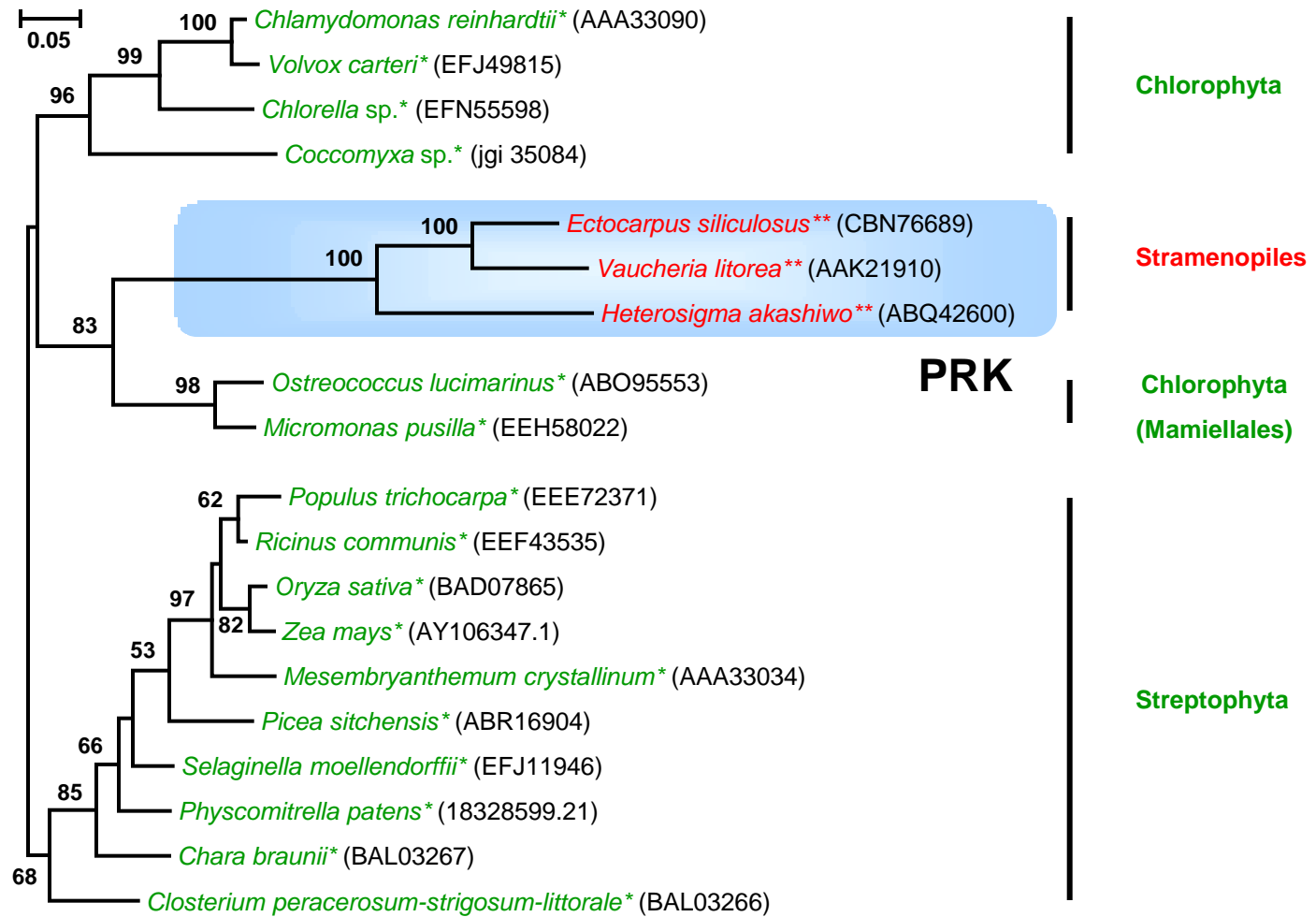

Figure S2c

Supplement: Supplementary Data [file supp_evu043_Petersen_FigS2.pdf]

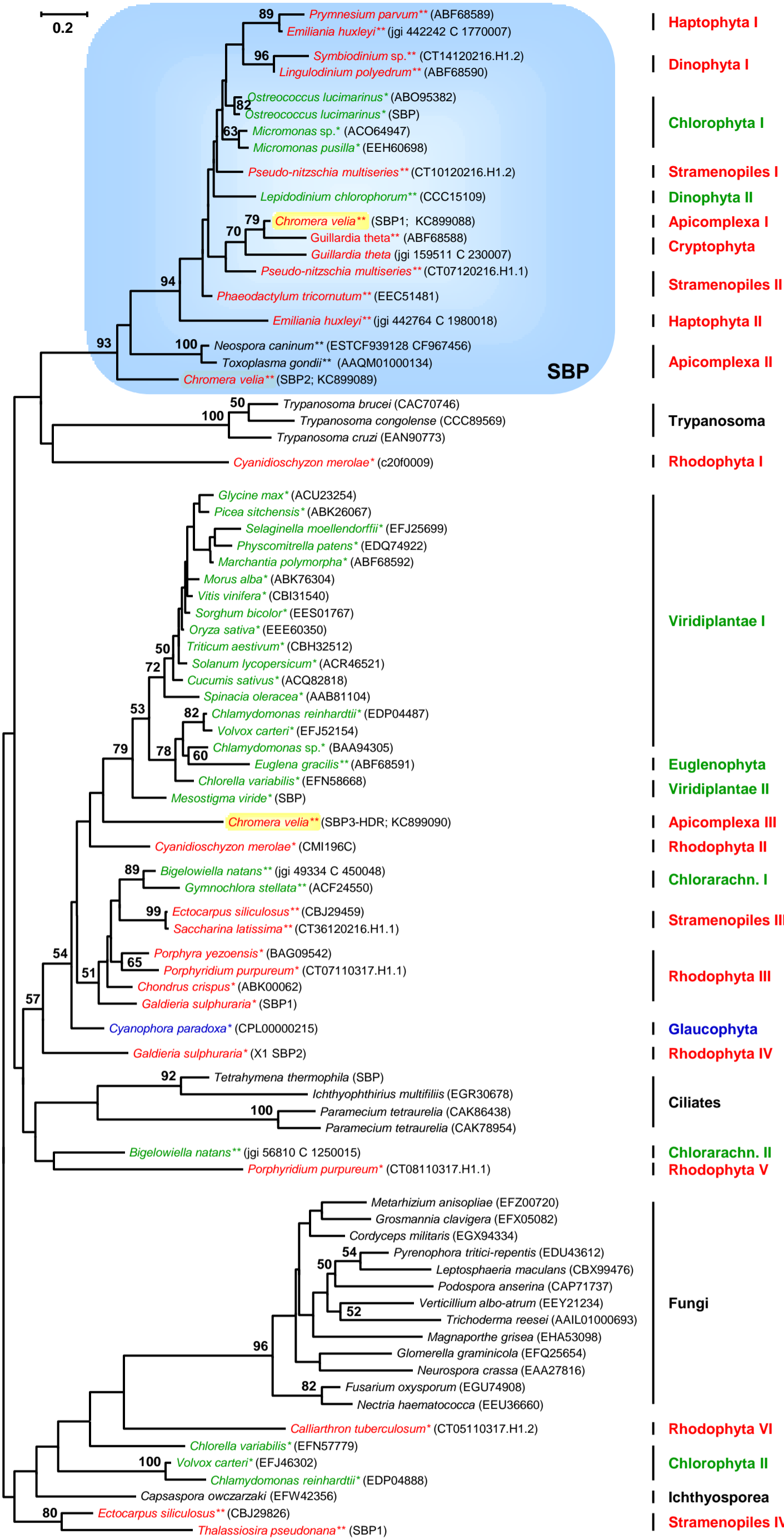

Figure S3a

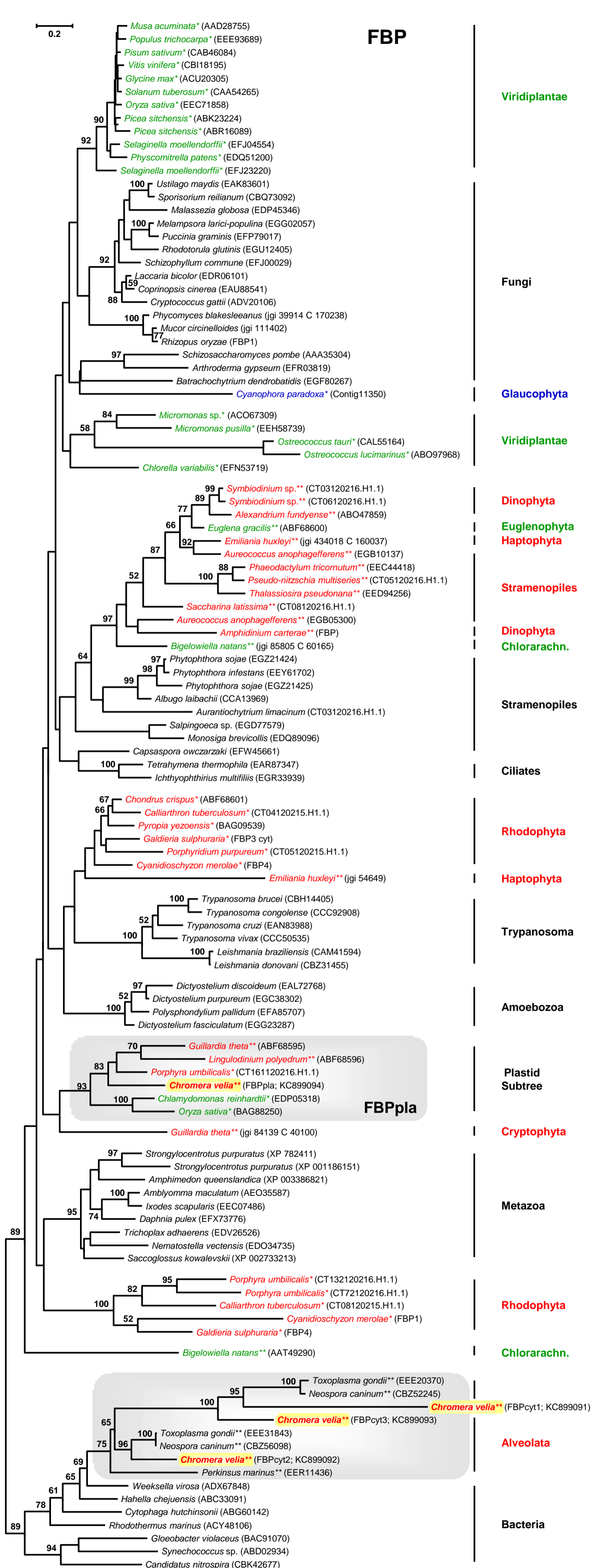

Figure S3b

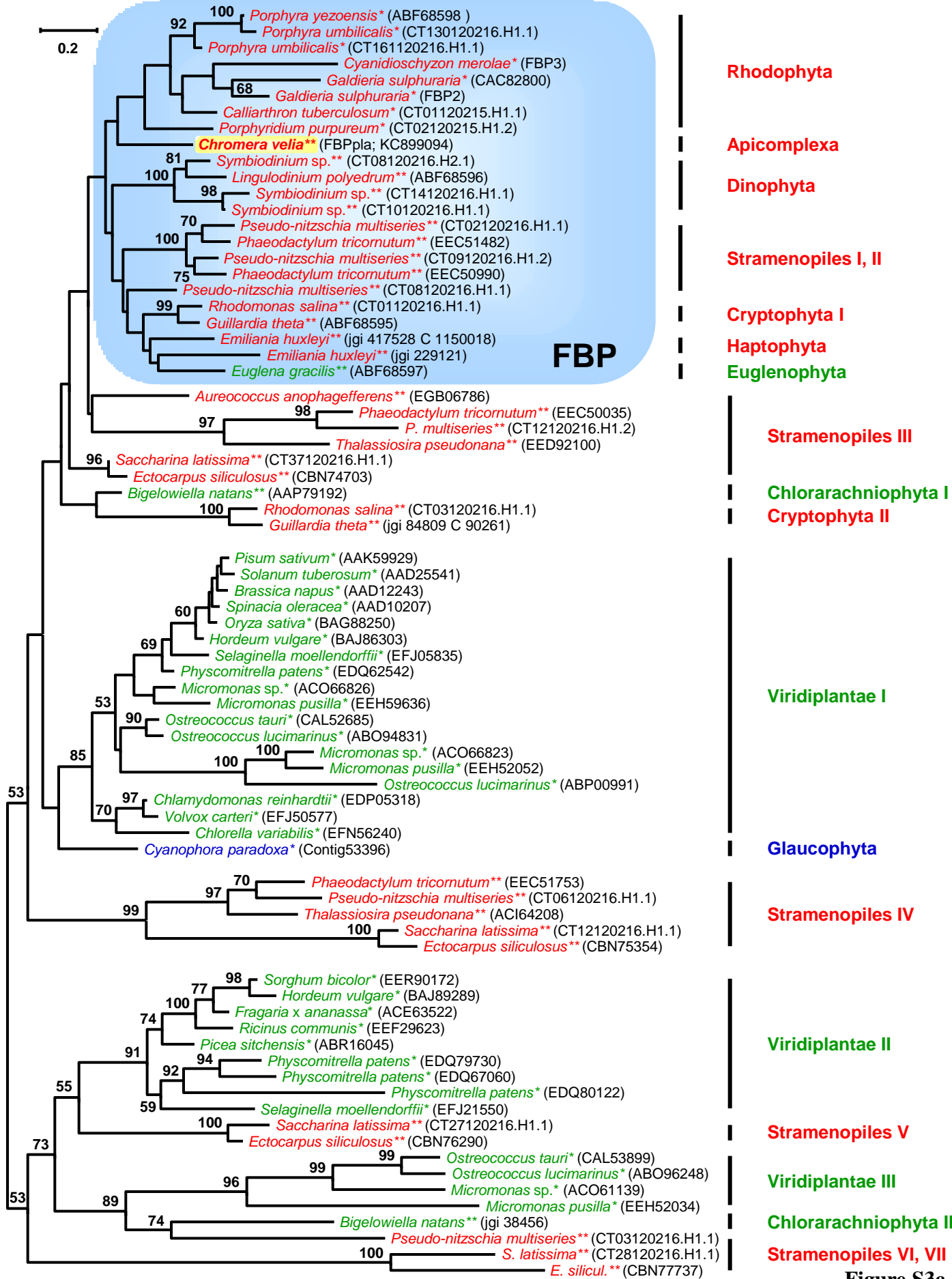

Figure S3c

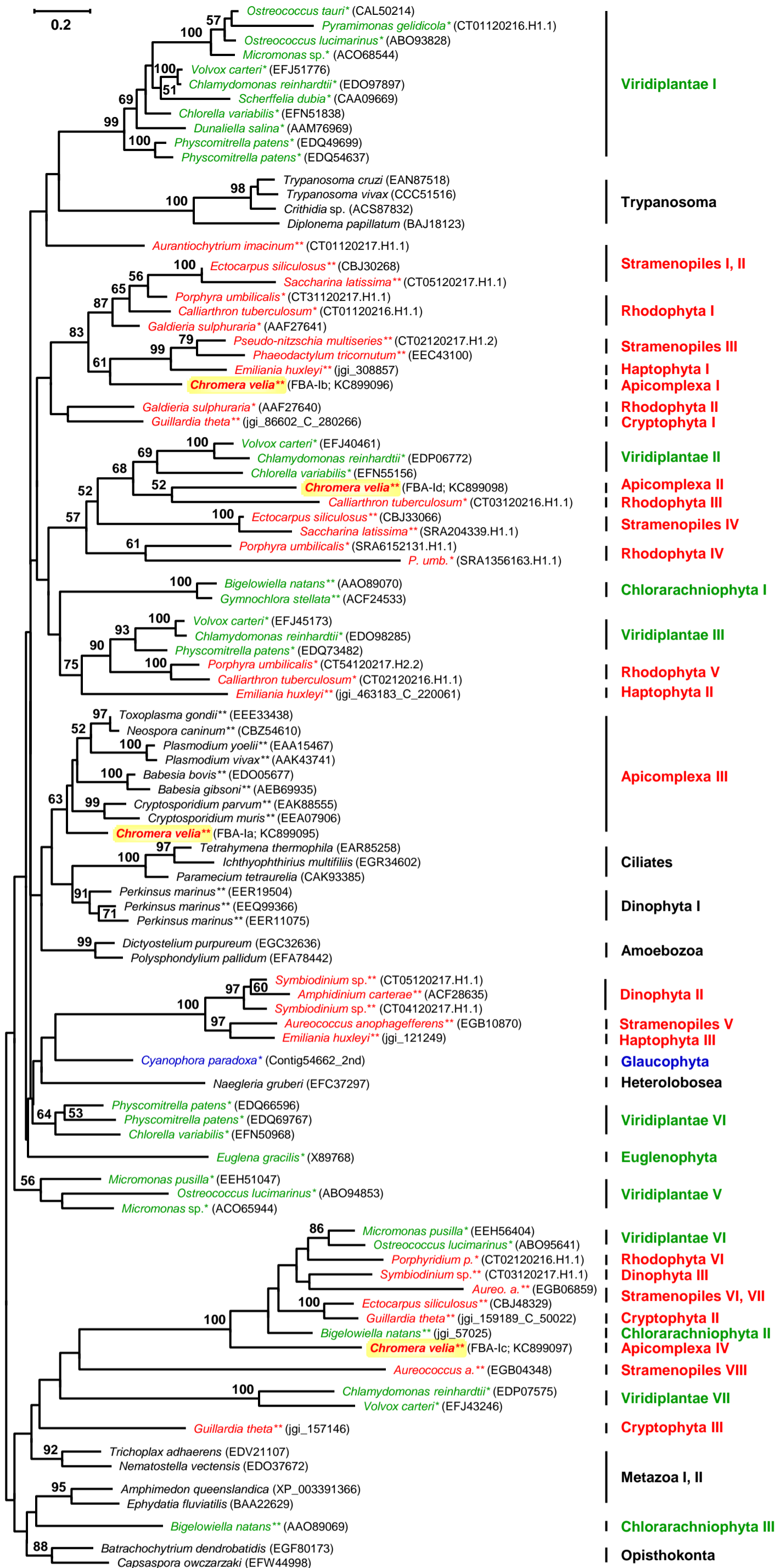

Figure S3d

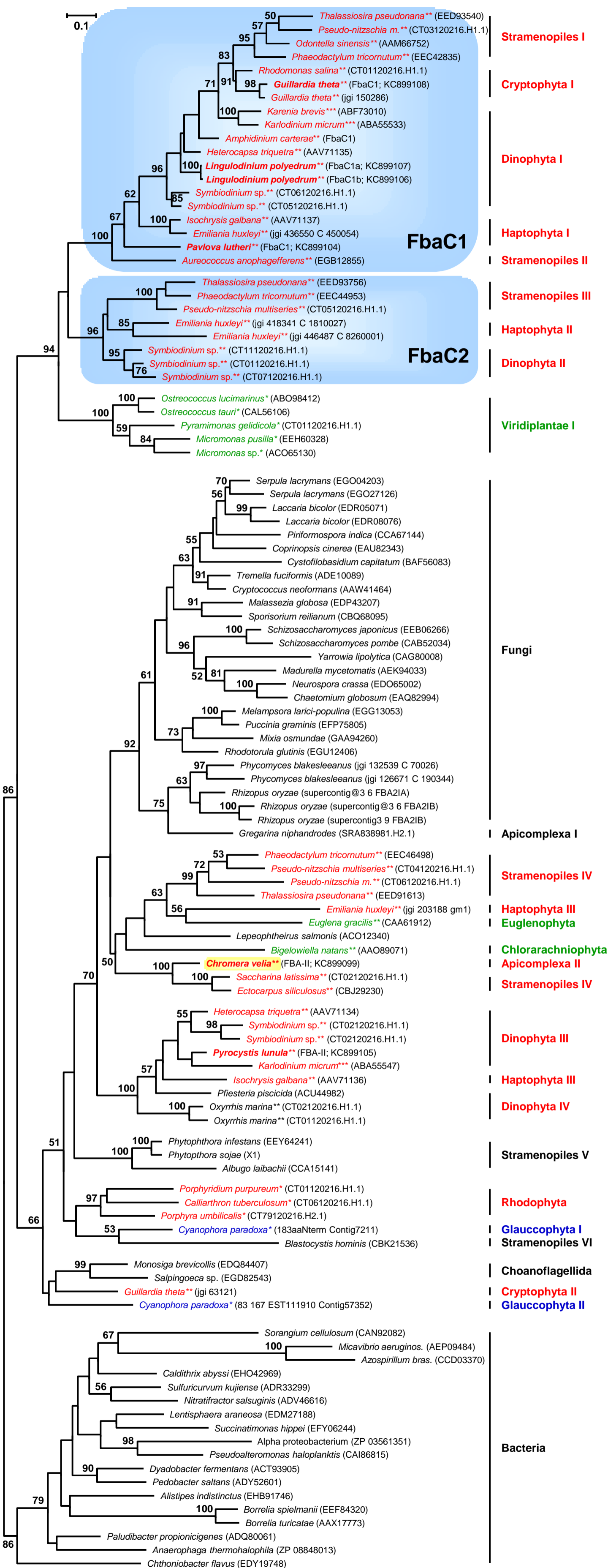

Figure S3e

Supplement: Supplementary Data [file supp_evu043_Petersen_FigS3.pdf]

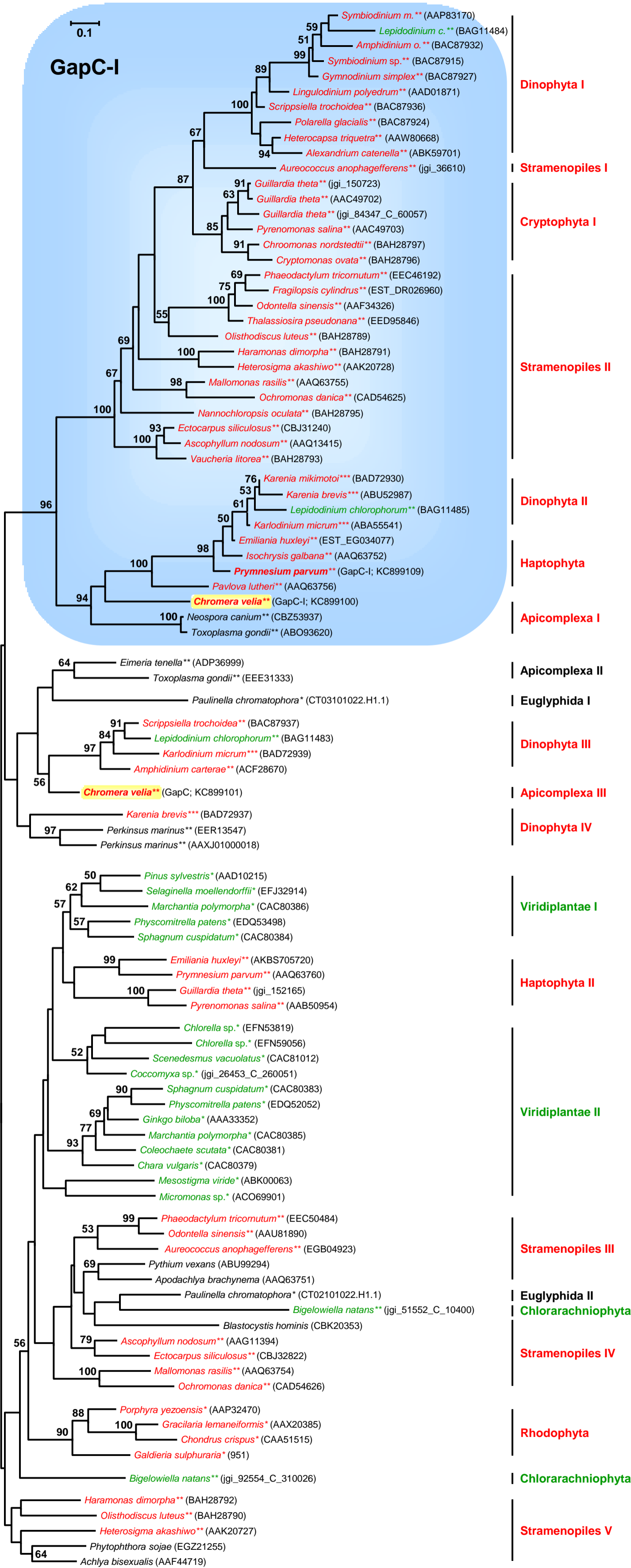

Figure S4

Supplement: Supplementary Data [file supp_evu043_Petersen_FigS4.pdf]

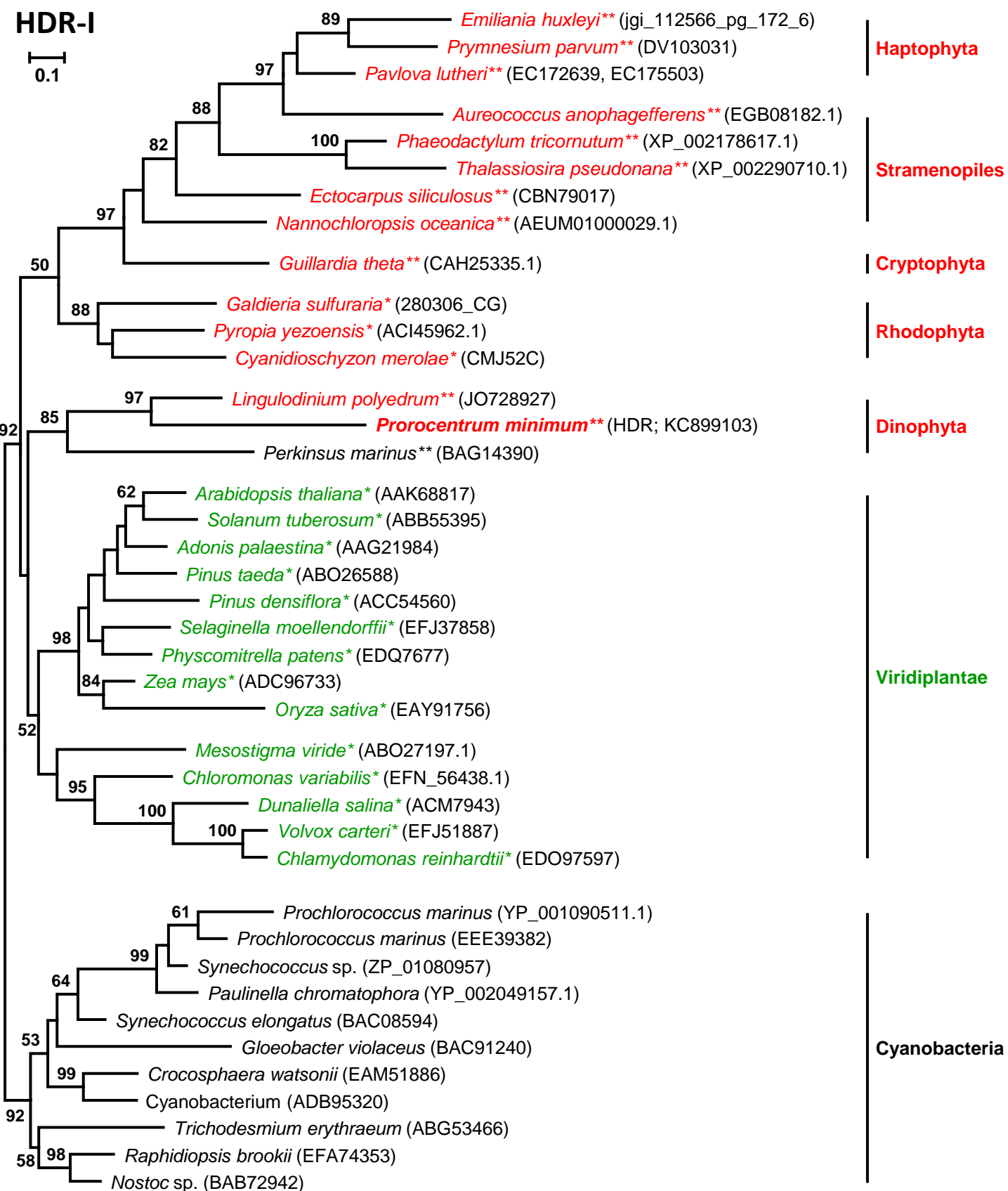

Figure S5a

HDR-II

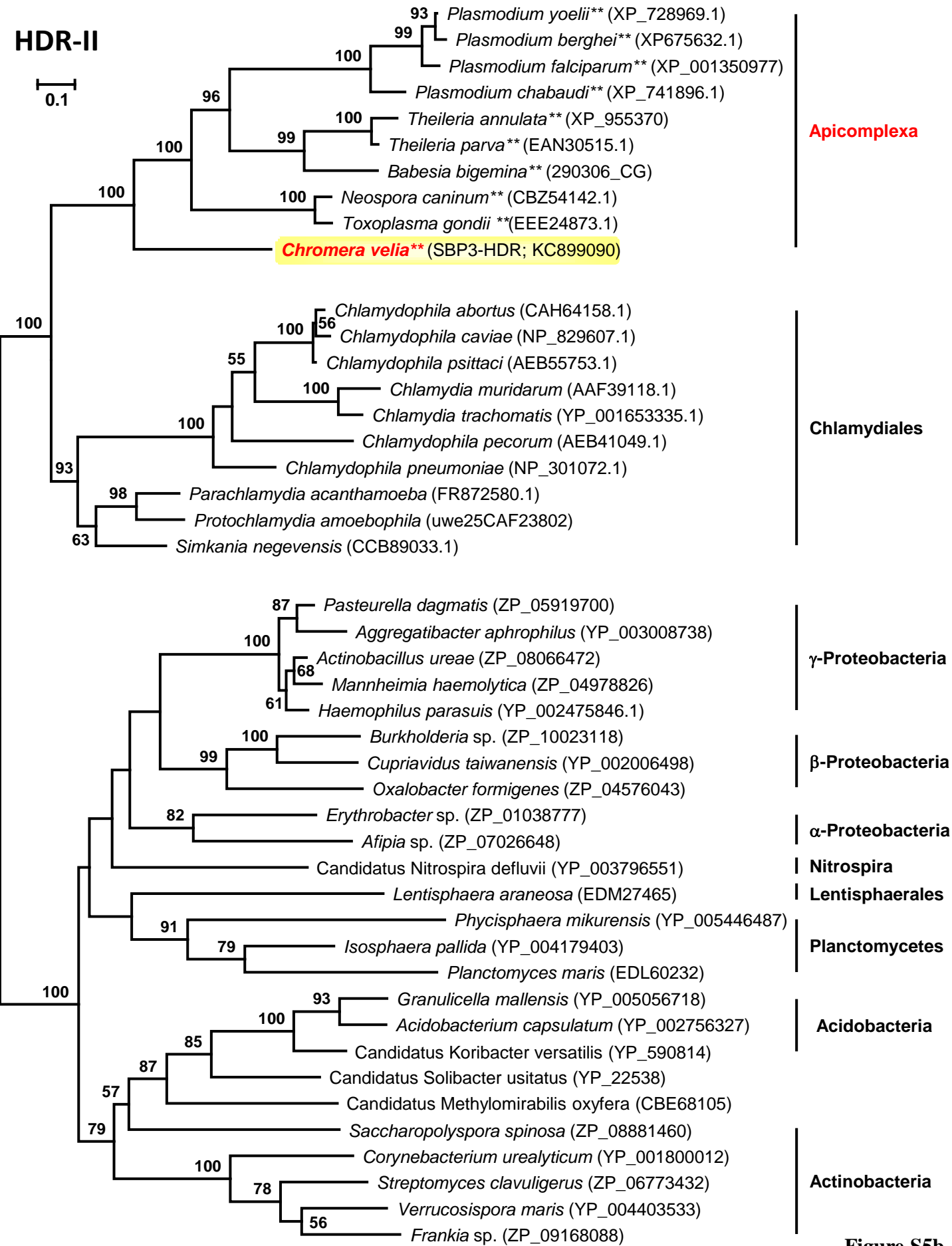

Figure S5b

Supplement: Supplementary Data [file supp_evu043_Petersen_FigS5.pdf]
