# Supplementary material for: Chromera velia, Endosymbioses and the Rhodoplex Hypothesis—Plastid Evolution in Cryptophytes, Alveolates, Stramenopiles, and Haptophytes (CASH Lineages)
Source: Genome Biol Evol. 2014 Feb 25;6(3):666–84. doi: 10.1093/gbe/evu043 (PMC3971594; doi:10.1093/gbe/evu043)
Supplement: Supplementary Data [file supp_evu043_Petersen_FigS6neu.pdf]

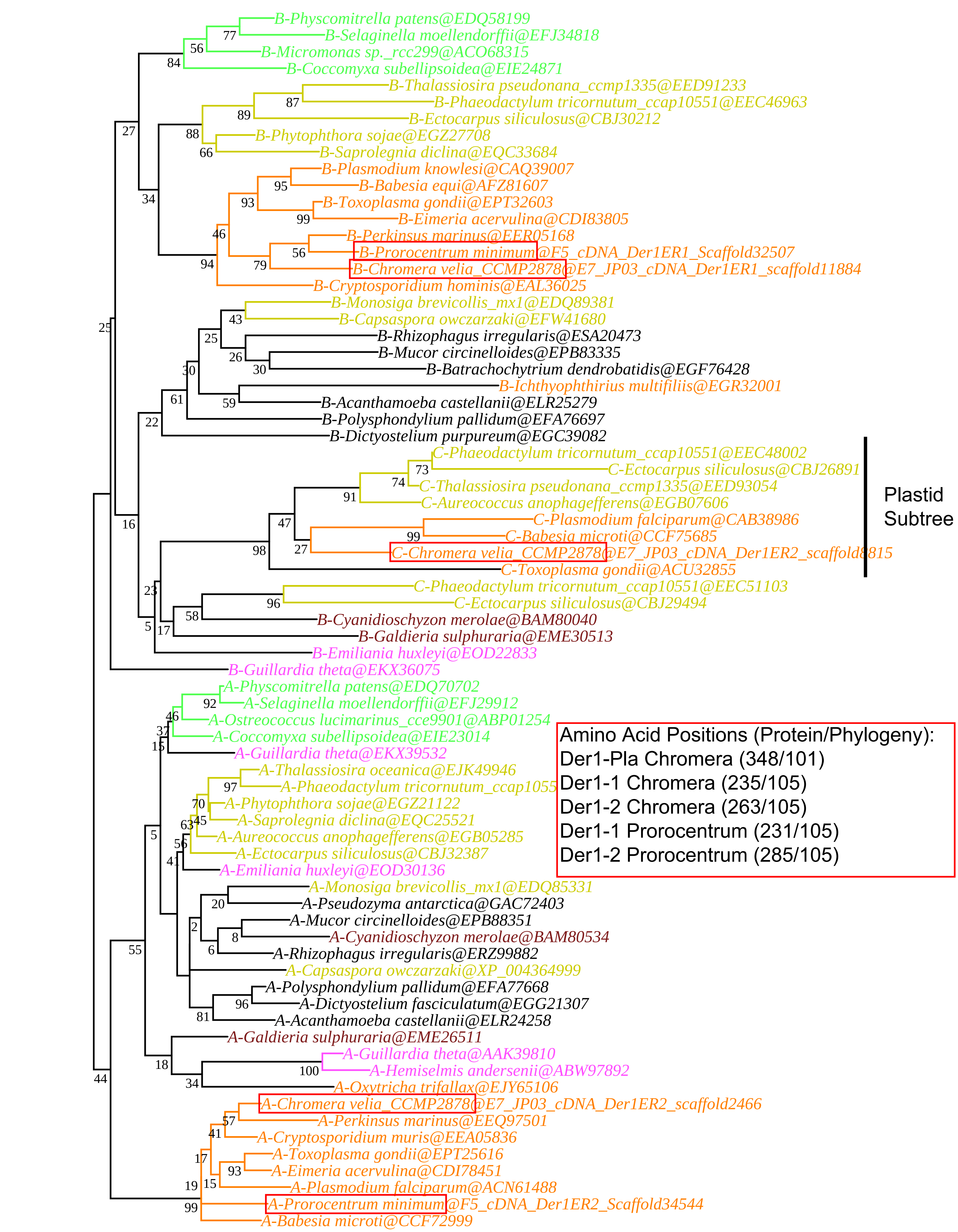

Supplemental Figure S6a: Phylogenetic Maximum Likelihood analysis of Der1 proteins

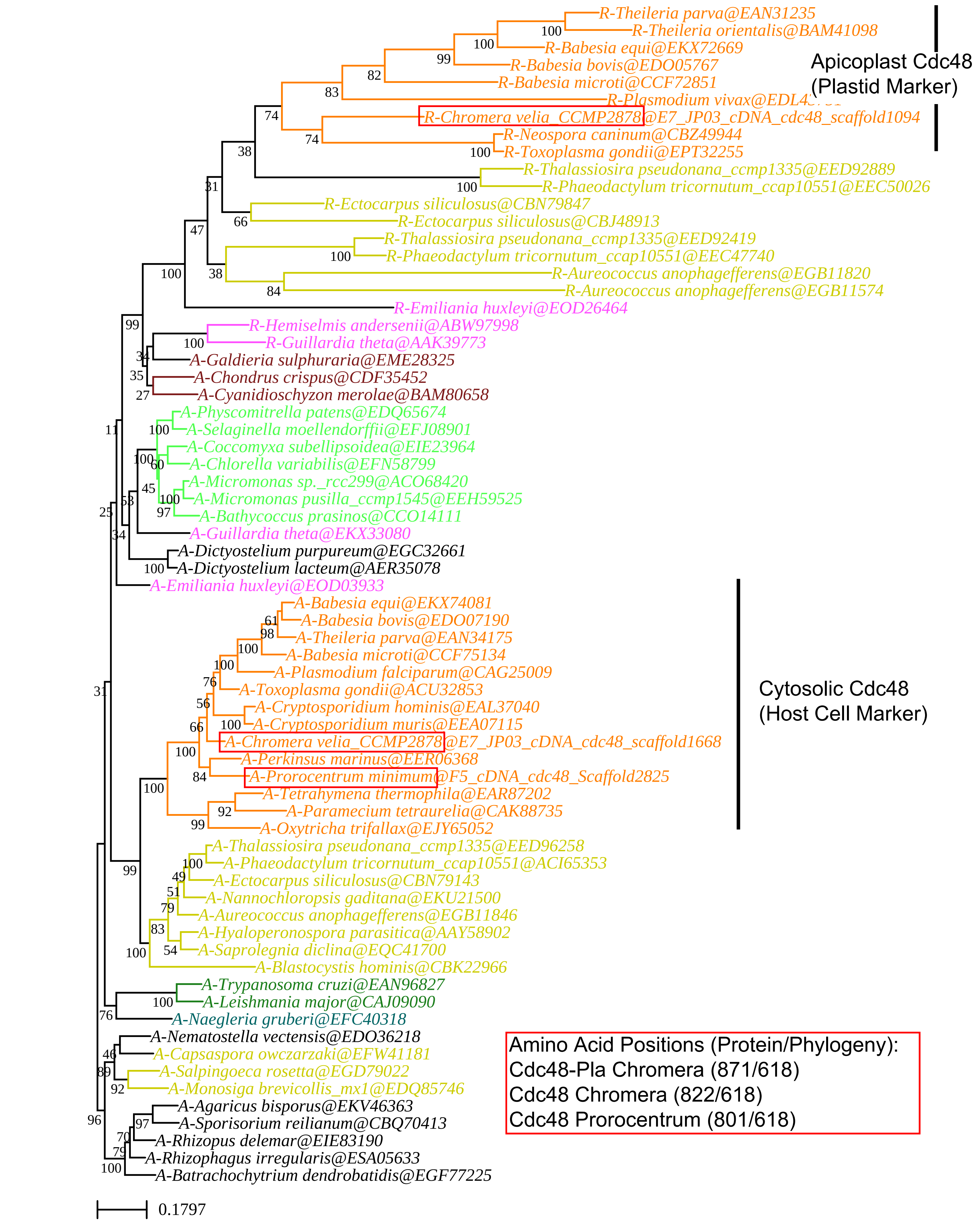

Supplemental Figure S6b: Phylogenetic Maximum Likelihood analysis of Cdc48 proteins

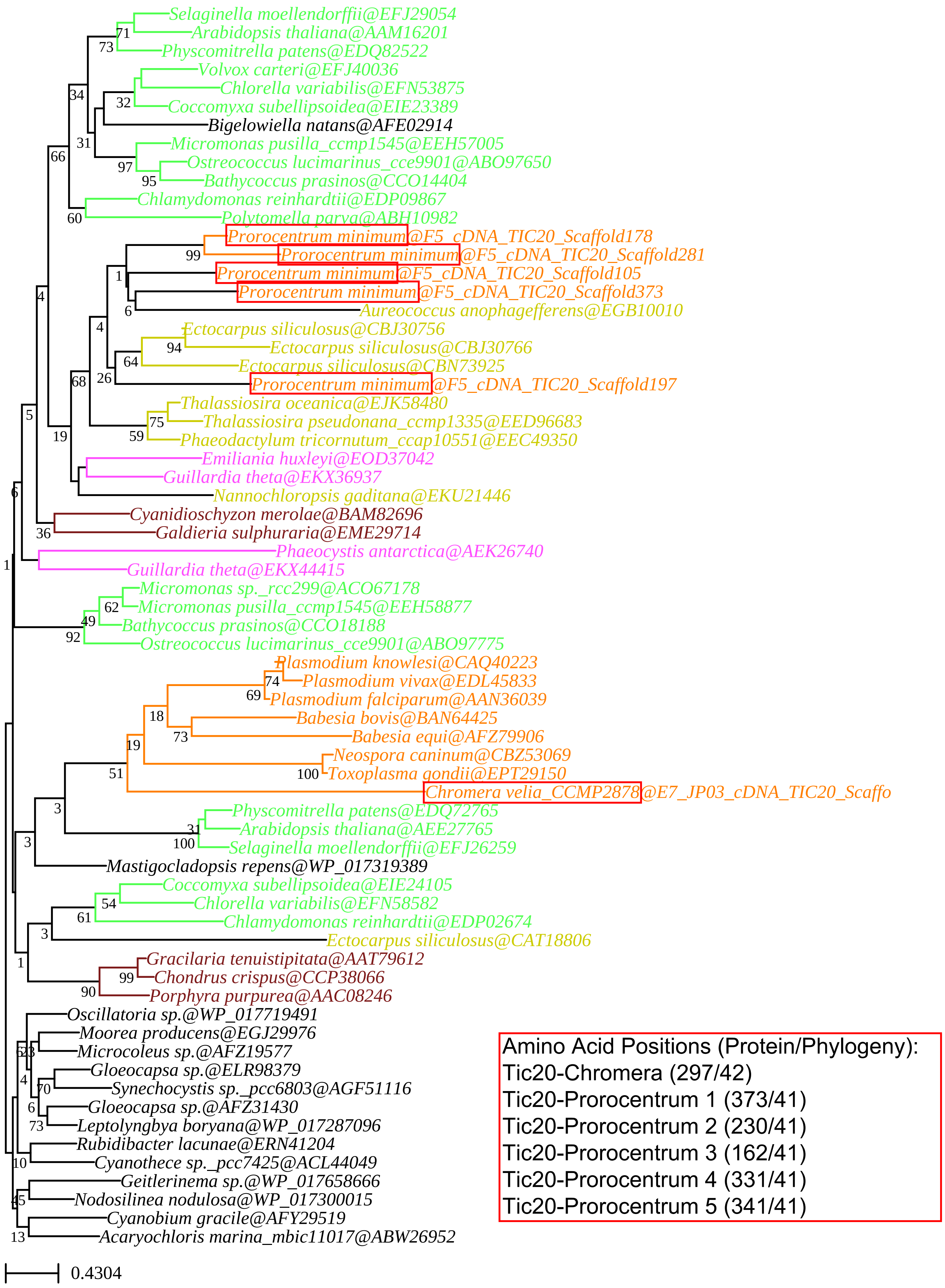

Supplemental Figure S6c: Phylogenetic Maximum Likelihood analysis of Tic20

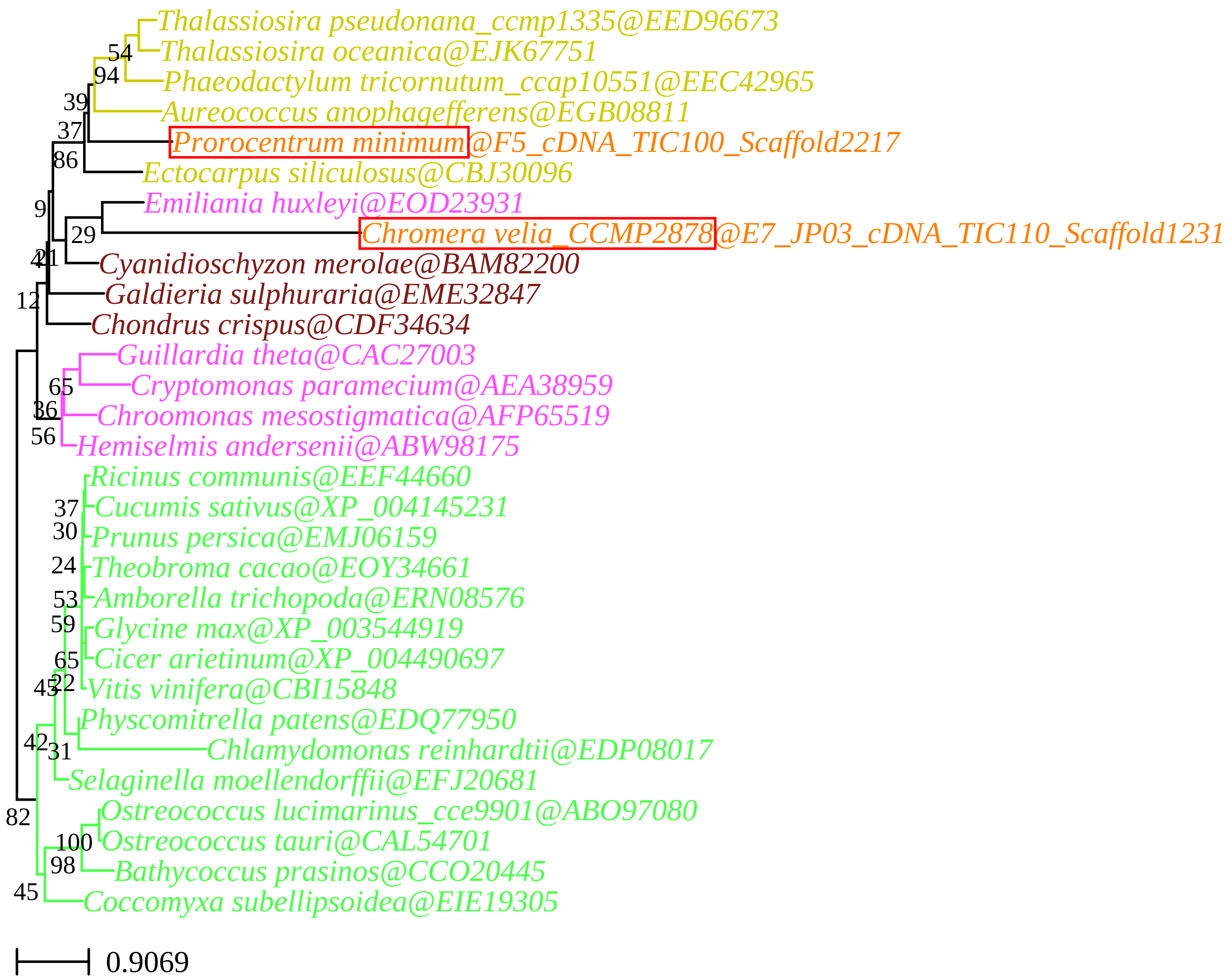

Amino Acid Positions (Protein/Phylogeny):
Tic110-Chromera (1174AA/111AA)
Tic110-Prorocentrum (936AA/110AA)

Supplemental Figure S6d: Phylogenetic Maximum Likelihood analysis of Tic110
