# Supplementary material for: Chromera velia, Endosymbioses and the Rhodoplex Hypothesis—Plastid Evolution in Cryptophytes, Alveolates, Stramenopiles, and Haptophytes (CASH Lineages)
Source: Genome Biol Evol. 2014 Feb 25;6(3):666–84. doi: 10.1093/gbe/evu043 (PMC3971594; doi:10.1093/gbe/evu043)
Supplement: Supplementary Data [file supp_evu043_Petersen_TabS1.pdf]

Supplemental Table S1: Comparison of Illumina, 454 and Sanger sequence coverage exemplified for 16 cDNAs from *Chromera velia* CCAP 1602/1

				**Illumina Approach** (current study)				**454 Approach** (Woehle et al. 2011)				**Sanger Approach** (unpublished)			
No	Gene	Introns	Coverage	Accession	Coverage	Length	Protein	Accession*	TSAs	Length	Protein	Accession*	ESTs	Length	Protein
1	PRK	10	19.2x	KC899087	755x	2,767 bp	full length	JO788081.1	1	1,846 bp	partial	HO866516.1	3	845 bp	partial
2	SBP1	1	17.5x	KC899088	1,999x	2,258 bp	full length	JO790107.1	2	1,449 bp	partial	-	-	-	-
3	SBP2	4	18.4x	KC899089	155x	1,225 bp	full length	-	-	-	-	-	-	-	-
4	SBP3-HDR	15	19.5x	KC899090	505x	3,275 bp	full length	JO802140.1	1	1,132 bp	partial[3]	-	-	-	-
5	FBPcyt1	7	17.2x	KC899091	146x	2,172 bp	full length	JO798440.1	2	2,142 bp	full length	-	-	-	-
6	FBPcyt2	9	18.1x	KC899092	356x	2,513 bp	full length	JO800087.1	2	1,267 bp	partial	-	-	-	-
7	FBPcyt3	7	18.8x	KC899093	321x	2,801 bp	full length	JO788854.1	3	805 bp	partial	HO866483.1	3	799 bp	partial
8	FBPpla	11	18.8x	KC899094	13,563x	3,364 bp	full length	JO790611.1	5	760 bp	partial	HO866945.1	2	820 bp	partial
9	FBA-I a	9	16.5x	KC899095	1,243x	1,600 bp	full length	JO801881.1	1	865 bp	partial	-	-	-	-
10	FBA-I b	4	17.6x	KC899096	1,966x	1,996 bp	full length	JO795142.1	1	1,438 bp	full length	HO866136.1	3	711 bp	partial
11	FBA-I c	9	19.6x	KC899097	105x	1,615 bp	full length[1]	-	-	-	-	-	-	-	-
12	FBA-I d	0	17.3x	KC899098	111x	1,325 bp	full length[2]	JO787575.1	1	1,544 bp	full length	-	-	-	-
13	FBA-II	2	19.3x	KC899099	1,976x	2,647 bp	full length	JO805056.1	2	606 bp	partial	HO866767.1	2	829 bp	partial
14	GapC-I	0	20.2x	KC899100	816x	1,994 bp	full length	JO786707.1	1	1,989 bp	full length	HO865964.1	4	843 bp	full length[1]
15	GapC	4	17.0x	KC899101	2,598x	1,703 bp	full length	JO789461.1	2	1,650 bp	partial	HO866153.1	7	863 bp	partial
16	Not1	32	17.6x	KC899102	307x	13,382 bp	full length	JO802605.1	1	928 bp	partial	-	-	-	-

* largest transcriptome shotgun assembly (TSA) or expressed sequence tag (EST); [1] cDNA merged from two overlapping TSAs; [2] cDNA merged from three TSAs in comparison with genomic contigs; [3] N-terminal SBP3 sequence not covered, C-terminal HDR sequence partial.
